# Broadband generation of perfect Poincaré beams via dielectric spin-multiplexed metasurface

**DOI:** 10.1038/s41467-021-22462-z

**Published:** 2021-04-13

**Authors:** Mingze Liu, Pengcheng Huo, Wenqi Zhu, Cheng Zhang, Si Zhang, Maowen Song, Song Zhang, Qianwei Zhou, Lu Chen, Henri J. Lezec, Amit Agrawal, Yanqing Lu, Ting Xu

**Affiliations:** 1 National Laboratory of Solid-State Microstructures, Jiangsu Key Laboratory of Artificial Functional Materials, College of Engineering and Applied Sciences, Nanjing University, Nanjing, China; 2 Collaborative Innovation Center of Advanced Microstructures, Nanjing, China; 3 Physical Measurement Laboratory, National Institute of Standards and Technology, Gaithersburg, MD USA; 4 Maryland NanoCenter, University of Maryland, College Park, MD USA; 5 School of Optical and Electronic Information, Wuhan National Laboratory for Optoelectronics, Huazhong University of Science and Technology, Wuhan, China

**Keywords:** Metamaterials, Sub-wavelength optics

## Abstract

The term Poincaré beam, which describes the space-variant polarization of a light beam carrying spin angular momentum (SAM) and orbital angular momentum (OAM), plays an important role in various optical applications. Since the radius of a Poincaré beam conventionally depends on the topological charge number, it is difficult to generate a stable and high-quality Poincaré beam by two optical vortices with different topological charge numbers, as the Poincaré beam formed in this way collapses upon propagation. Here, based on an all-dielectric metasurface platform, we experimentally demonstrate broadband generation of a generalized perfect Poincaré beam (PPB), whose radius is independent of the topological charge number. By utilizing a phase-only modulation approach, a single-layer spin-multiplexed metasurface is shown to achieve all the states of PPBs on the hybrid-order Poincaré Sphere for visible light. Furthermore, as a proof-of-concept demonstration, a metasurface encoding multidimensional SAM and OAM states in the parallel channels of elliptical and circular PPBs is implemented for optical information encryption. We envision that this work will provide a compact and efficient platform for generation of PPBs for visible light, and may promote their applications in optical communications, information encryption, optical data storage and quantum information sciences.

## Abstract (web summary)

Well-controlled Poincaré beams are potentially useful in optical communications applications. Here, the authors present phase-only metasurfaces that generate broadband, perfect Poincaré beams in the visible, with radius independent of the topological number.

## Introduction

Polarization and phase are two intrinsic properties of light^1^. Light can possess a spin angular momentum (SAM) of *σћ* (*σ* = ± 1) per photon depending on the chirality of circular polarization^2^, and an orbital angular momentum (OAM) of *lћ* (where *l* is the topological charge number) per photon depending on its helical phase structure^3^. SAM and OAM constitute the total angular momentum (*J* = *σћ* *+* *lћ*) of a light beam^4^. The hybrid-order Poincaré Sphere (HyOPS)^5^, which generalizes Poincaré Sphere (PS)^6^ and higher-order Poincaré Sphere (HOPS)^7^, has been developed to describe the total angular momentum and the evolution of polarization and phase of light. The poles of the HyOPS denote two orthogonal, circularly polarized states of optical vortices^8^ with arbitrary topological charge numbers. Each point on the HyOPS describes the space-variant polarization field of a light beam carrying OAM, which can be represented by a linear superposition of the two poles and is generally referred to as Poincaré beam^9^. In particular, the optical vortices and cylindrical vector vortex beams^10^ described by the poles and equator of the HyOPS, respectively, have been demonstrated and applied in a number of applications^11^, such as optical trapping, high-resolution microscopy, optical communication, nonlinear optics, and optical encryption^12^. However, the beam radii of these conventional Poincaré beams always depends on their topological charge number, hindering their applications in some cases, for e.g., coupling of multiple Poincaré beams into a single optical fiber for mode division multiplexing^13^. In addition, it is difficult to generate a stable and high-quality Poincaré beam by two orthogonally circularly polarized optical vortices with very different topological charge numbers, as the Poincaré beam formed in this way would easily collapse upon propagation^14^.

Recently, the concept of perfect optical vortices (POVs) has been proposed to overcome the above limitations because their ring radii are independent of the topologic charges^15^. Consequently, perfect Poincaré beams (PPBs), as the linear superposition of POVs, have been demonstrated using selective combinations of conventional optical components, such as axicons, spatial light modulators, *q*-plates, and Fourier transform lenses^16–18^. However, the complex light path and bulky footprint of optical components generally required in these approaches make them non-ideal for integration into compact nanophotonic platforms. Furthermore, optical aberrations caused by misalignment between the different optical components can easily deteriorate the quality of PPBs. In addition, conventional spatial light modulators and *q*-plates have micron-scale sampling pixel sizes that significantly limits the spatial density of the generated PPBs array.

Metasurfaces, made up of nanoscale optical scatters, can arbitrarily modulate the phase, polarization, and amplitude of light with deep subwavelength spatial resolution, and thus provides an efficient, versatile, and integration-friendly platform for compact planar optics^19–30^. In particular, due to the flexible design freedom offered by them, transmissive metasurfaces have been applied to a wide variety of functional planar optics, such as imaging lenses^31–35^, metaholograms^36–38^, nonlinear optics^39,40^, and structured beam generators^41–43^. Recently, a plasmonic metasurface was shown to generate POVs^44^; however, it suffers from low efficiency due to the large ohmic absorption caused by the constituent metallic nanostructures. A silicon metasurface has also been designed to generate PPBs^45^; however, this design requires simultaneous phase and amplitude modulation and a complicated oblique incidence scheme, which further limit its efficiency and operating bandwidth.

In this work, based on an all-dielectric metasurface, we propose and experimentally demonstrate a compact platform for broadband generation of generalized PPBs, including both elliptical and circular shapes. Through phase-only modulation, a single-layer metasurface composed of subwavelength-spaced titanium dioxide (TiO_2_) nanopillars can straightforwardly achieve all the states of PPBs on the HyOPS for visible light, without the requirement of any additional optical elements. By combining both geometric phase and propagation phase modulation^46^, the designed spin-multiplexed metasurface can provide two arbitrary, fully uncorrelated phase profiles for the two orthogonal circular polarizations of incident light. By changing the polarization states of incident light, all orders of PPBs with arbitrary phase and polarization distributions can be generated. Furthermore, as a proof-of-concept demonstration, a metasurface encoding multidimensional SAM and OAM states in parallel channels for elliptical and circular PPBs is implemented for optical information encryption. We envision that this work will provide a platform for the efficient generation of PPBs for visible light and may promote their applications in optical communications, information encryption, optical data storage, and quantum information science. Furthermore, this method to achieve complete and independent phase control of orthogonal circular polarization states at the single pixel level also can be used to design vectorial metasurface to generate arbitrary 3D vectorial field distribution for 3D vectorial holography application^47^. This vectorial metasurface may promote the development of compact and highly-integrated optical system for polarization holography, Stokes holography, holographic trap display, multidimensional data storage, and optical microscopic imaging.

## Results

### Design of metasurface to generate generalized PPBs

**Fig. 1:**
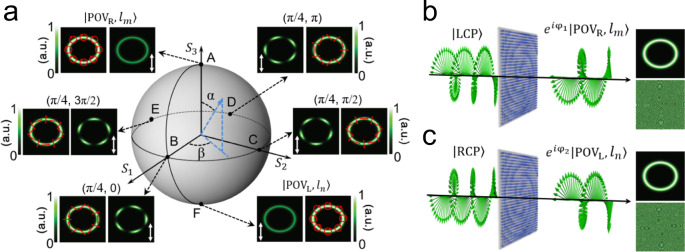
Principle of generation of generalized perfect Poincaré beams (PPBs) via dielectric metasurface. **a** A hybrid-order Poincaré Sphere (HyOPS) representation of various PPBs. As an example, the two poles are represented by two perfect vortices (POVs) with the same ellipticity and different topological charges *l*_*m*_ = 5 and *l*_*n*_ = 10. The annular intensity profiles of six PPBs (red arrows represent the polarization distributions) with different coordinates are of same size and these elliptical hollow beams are transformed into distinct patterns using a vertical linear polarizer depicted by the white double arrow. **b**, **c** Left: schematic illustration of the metasurface capable of providing two independent phase profiles φ_1_ and φ_2_ for LCP and RCP light, respectively. The output beam becomes a RCP (**b**) or LCP (**c**) POV with the topological charge of *l*_*m*_ (**b**) or *l*_*n*_ (**c**). Right: an example of the intensity (top) and phase (bottom) profiles of metasurface-generated POV with *l*_*m*_ = 5 (**b**) and *l*_*n*_ = 10 (**c**).

The vectorial field of a monochromatic paraxial light beam can be represented by the superposition of scalar fields with orthogonal polarization states^11^. Considering the orthogonal circular polarization basis and two POVs with same ring radius but different topological charges, a PPB can be described as:

1 $$|{U}_{N}\rangle =\,\cos \left(\frac{\alpha }{2}\right){e}^{i\beta /2}|{{\rm{POV}}}_{{\mathrm{R}}},{l}_{m}\rangle +\,\sin \left(\frac{\alpha }{2}\right){e}^{-i\beta /2}|{{\rm{POV}}}_{{\mathrm{L}}},{l}_{n}\rangle$$

where $|{{\rm{POV}}}_{{\mathrm{R}}},{l}_{m}\rangle$ and $|{{\rm{POV}}}_{{\mathrm{L}}},{l}_{n}\rangle$ denote the right-handed circularly polarized (RCP) and left-handed circularly polarized (LCP) POV with same ellipticity, and different topological charge numbers of *l*_*m*_ and *l*_*n*_, respectively. $\cos (\frac{\alpha }{2})$ and $\sin (\frac{\alpha }{2})$ represent the amplitude of RCP and LCP POV and *β* is the relatively phase difference between them, where *α* ∈ [0, π] and *β* ∈ [0, 2π]. $|{U}_{N}\rangle$ is an arbitrary point on the surface of HyOPS with spherical coordinates (*α*, *β*). Figure 1a shows examples of various elliptical PPBs and their mapping points on the HyOPS. Each point on the surface of the HyOPS denotes a state of a PPB with space-variant polarization and phase distribution. The polarization distribution of each state can be determined by polarization order $p=({l}_{m}-{l}_{n})/2$ and the HyOPS spherical coordinates (*α*, *β*). The phase distribution of the PPB is characterized by the topological Pancharatnam charge ${l}_{p}=({l}_{m}+{l}_{n})/2$^14,45^. Two poles of the HyOPS are represented by two elliptical POVs with same ellipticity factor *γ* = 1.2 and uniform circular polarization (depicted by red arrows) but different topological charge numbers *l*_*m*_ = 5 and *l*_*n*_ = 10. The annular intensity profiles at other points on the HyOPS are also of the same ring radius; and due to their anisotropic linear polarization distributions (depicted by red arrows), these elliptical hollow beams can be transformed to exhibit distinct intensity patterns by using a vertical linear polarizer (indicated by the white double arrow).

In principle, a POV can be generated by taking the Fourier transform of a higher-order Bessel beam^15^. Due to the difficulty of experimentally generating an ideal Bessel beam, here we consider the Fourier transform of a higher-order Bessel-Gaussian beam instead. To be specific, a Gaussian beam can be transformed into a POV by passing it through a spiral phase plate, an axicon, and a Fourier transform lens. In terms of the Fourier transform theory, the complex field amplitude of the POV with uniform polarization in the rear focal plane of a lens can be obtained as (see Supplementary Note 1 for details):

2 $${\boldsymbol{E}}(r,\varphi )=\frac{{\omega }_{g}{i}^{l-1}}{{\omega }_{\gamma }}\exp (il\varphi )\exp \left(\frac{-{(r-{R}_{\gamma })}^{2}}{{\omega }_{\gamma }^{2}}\right)\left[\begin{array}{c}1\\ \pm i\end{array}\right]$$

where (*r*, *φ*) are the polar coordinates, *ω*_*g*_ is the waist of the input Gaussian beam, *ω*_*γ*_ is the waist of the Gaussian beam in the rear focal plane, and *l* is the topological charge. ${R}_{\gamma }=\gamma {k}_{r}f/k$, where *k*_*r*_ is the radial wavevector relying on the numerical aperture (NA) of the axicon, $k=2\pi /{\lambda }_{0}$ is the free-space wavevector, and *f* is the focal length of lens. It is obvious that the amplitude of the POV is shaped by a Gaussian function, which has a maximum value at *r* = *R*_*γ*_. The radii of the POV along the vertical and horizontal directions can be expressed as *R*_⊥_ = *f*NA and *R*_||_ = *γf*NA, respectively, which are independent of topological charges. Therefore, depending on the values of three parameters (*γ*, *f*, NA), the intensity profile and radius along the major and minor axis of POV can be arbitrarily controlled. Therefore, customized shaping of the POV guarantees the generation of arbitrary annular intensity distribution of the PPB, providing sufficient variables for potential use in optical information encryption.

Due to the unique capability of flexible manipulation of the properties of light, a metasurface is able to integrate multiple functions of different optical elements, such as the spiral phase plate, axicon and Fourier transform lens as mentioned above, into a single monolithic device for PPB generation. For a polarized light beam normally incident onto the metasurface, it can be decomposed into LCP and RCP spin eigenstates corresponding to 2D Jones vectors: $$|{\rm{LCP}}\rangle =[\begin{array}{c}1\\ i\end{array}]$$ and $$|{\rm{RCP}}\rangle =[\begin{array}{c}1\\ -i\end{array}]$$. In order to generate two completely different POVs, the spin-multiplexed metasurface is required to provide two independent spatial phase profiles ${\varphi }_{1}(x,y)$ and ${\varphi }_{2}(x,y)$, respectively corresponding to $|{\rm{LCP}}\rangle$ and $|{\rm{RCP}}\rangle$ state (see Supplementary Note 2 for details). That is, for LCP incident light, the metasurface implements the transformation: $|{\rm{LCP}}\rangle \to |{{\rm{POV}}}_{{\mathrm{R}}},{l}_{m}\rangle$ (Fig. 1b). The output beam $|{{\rm{POV}}}_{{\mathrm{R}}},{l}_{m}\rangle$ has flipped handedness compared to the incident light. Similarly, for the RCP incident light, the metasurface transforms it to a different POV with LCP state and topological charge of *l*_*n*_: $|{\rm{RCP}}\rangle \to |{{\rm{POV}}}_{{\mathrm{L}}},{l}_{n}\rangle$ (Fig. 1c). Therefore, the metasurface can be described by a Jones matrix *J*(*x*, *y*) which simultaneously satisfies $J(x,\,y)|{\rm{LCP}}\rangle =\exp [i{\varphi }_{1}(x,y)]|{\rm{RCP}}\rangle$ and $J(x,y)|{\rm{RCP}}\rangle =\exp [i{\varphi }_{2}(x,y)]|{\rm{LCP}}\rangle$. The required Jones matrix takes the form:

3 $$J(x,y)=\left[\begin{array}{lll}\frac{\exp [i{\varphi }_{1}(x,y)]+\exp [i{\varphi }_{2}(x,y)]}{2} & \frac{i\exp [i{\varphi }_{2}(x,y)]-i\exp [i{\varphi }_{1}(x,y)]}{2}\\ \frac{i\exp [i{\varphi }_{2}(x,y)]-i\exp [i{\varphi }_{1}(x,y)]}{2} & \frac{-\exp [i{\varphi }_{1}(x,y)]-\exp [i{\varphi }_{2}(x,y)]}{2}\end{array}\right]$$

Due to the symmetric and unitary conditions, *J*(*x*, *y*) can be written in a standard form *J*(*x*, *y*) = *RΛR*^−1^, where *R* is a real unitary matrix and *Λ* is a diagonal matrix. For birefringent optical elements constituting the metasurface, the diagonal matrix *Λ* determines their phase shifts *δ*_*x*_ and *δ*_*y*_ along the two perpendicular symmetry axes, while the matrix *R* determines the rotation angle *θ* of their fast axes relative to the reference coordinate in the *x*–*y* plane. (*δ*_*x*_, *δ*_*y*_), and *θ* determine the propagation phase and geometric phase imposed on the transmitted light, respectively. For the given spin-multiplexed phase profiles *φ*_1_(*x, y*) and *φ*_2_(*x, y*), the required phase shifts and rotation angle are calculated as (see Supplementary Note 3 for details):

4 $${\delta }_{x}(x,y)=[{\varphi }_{1}(x,y)+{\varphi }_{2}(x,y)]/2$$

5 $${\delta }_{y}(x,y)=[{\varphi }_{1}(x,y)+{\varphi }_{2}(x,y)]/2-\pi $$

6 $$\theta (x,y)=[{\varphi }_{1}(x,y)-{\varphi }_{2}(x,y)]/4$$

**Fig. 2:**
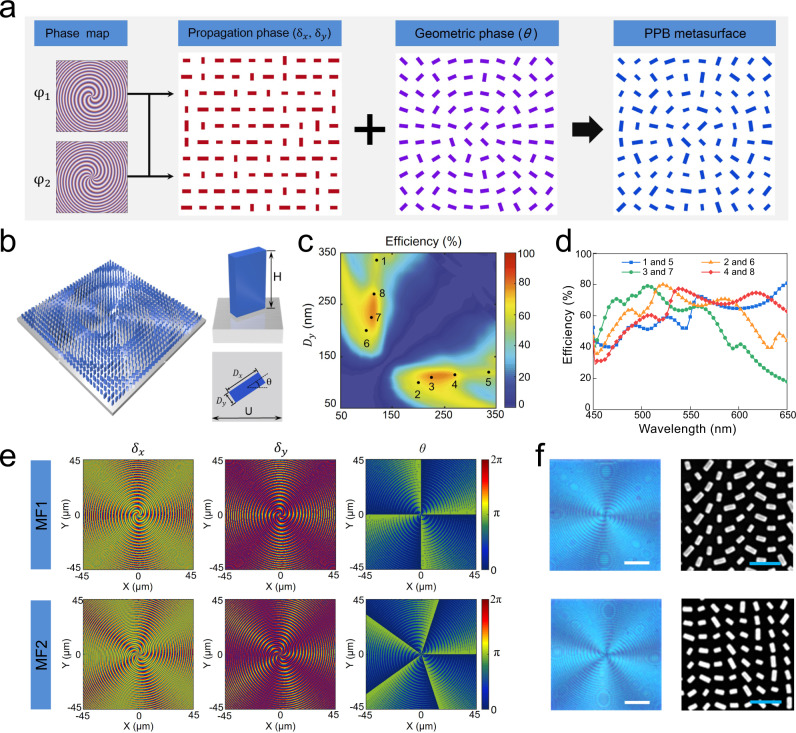
Design of a single-layer metasurface. **a** A general metasurface design method to generate arbitrary PPB. Given two arbitray phase map (*φ*_1_, *φ*_2_) for generation of different perfect vortices, the phase shifts (*δ*_*x*_, *δ*_*y*_) and rotation angle *θ* of metasurface pixels are calculated and utilized to design nanopillars with varying in-plane dimensions and orientation angle. **b** Left: schematic of the metasurface made up of TiO_2_ rectangle nanopillars. Right: perspective view and top view of the unit cell arranged on a fused-silica substrate. **c** Calculated polarization conversion efficiency as a function of nanopillars’ in-plane dimensions at a design wavelength of 530 nm. The black dots denote the selected nanopillars in constructing MF1 and MF2. **d** Calculated polarization conversion efficiencies of the selected eight nanopillars across the visible band. **e** The phase shifts (*δ*_*x*_, *δ*_*y*_) and rotation angle *θ* of the birefringent TiO_2_ nanopillars as a function of the spatial coordinates in the metasurface (MF1 and MF2) plane. **f** Left: optical microscope images of the fabricated metasurfaces: MF1 (top) and MF2 (bottom). Scale bar: 20 μm. Right: top view scanning electron microscopy (SEM) images of metasurfaces. Scale bar: 500 nm.

Therefore, in order to implement *J*(*x*, *y*), a series of subwavelength nanostructures are designed to provide the required phase shifts (*δ*_*x*_, *δ*_*y*_) covering the entire 2π phase range and satisfy the orientation angle *θ* at any point (*x*, *y*). The metasurface design flow is summarized in Fig. 2a. The propagation phase shifts *δ*_*x*_ and *δ*_*y*_ imparted by an individual nanopillar acting as a half-wave plate can be determined by its in-plane dimensions along the two perpendicular symmetry axes, and the geometric phase is controlled by orientation angle *θ* of the nanopillar. Combining them, the in-plane dimension and orientation of the nanopillars, of fixed height, constituting a vectorial metasurface can be determined at any point (*x*, *y*) on the metasurface plane.

Figure 2b shows a schematic diagram of metasurface composed of rectangular TiO_2_ nanopillars on a fused-silica substrate with a subwavelength square lattice constant *U*. The nanopillars are of uniform heights *H* while their in-plane dimensions (*D*_*x*_, *D*_*y*_), and orientation angles *θ* vary spatially. In order to investigate the transmission properties of rectangular TiO_2_ nanopillars, full-wave simulations are performed using the finite-difference time-domain (FDTD) technique. The height *H* of the TiO_2_ nanopillars is set as 600 nm to achieve the desired 2π phase coverage. The lattice constant *U* is chosen to be 450 nm, which guarantees that the nanopillars array act as a zeroth-order grating^22^ with relatively high transmission in the visible range. Supplementary Fig. 1 shows the simulated phase shifts and transmission coefficients of *x*- and *y*-polarized light for the rectangular nanopillars as a function of diameters (*D*_*x*_, *D*_*y*_) at a wavelength of *λ*_0_ = 530 nm. Based on these simulation results, polarization conversion efficiencies of TiO_2_ nanopillars are calculated and shown in Fig. 2c (see Supplementary Note 4 for details). A set of eight nanopillars, including four basic structures and four mirror structures are selected to provide eight phase levels covering the 2π phase range for *δ*_*x*_ and *δ*_*y*_ (Supplementary Fig. 2). The power transmission efficiency (*T*_*x*_ and *T*_*y*_) and phase shifts (*P*_*x*_ and *P*_*y*_) of eight nanopillars across the visible region are shown in Supplementary Fig. 3. As shown in Fig. 2d, these geometrical parameters (Supplementary Table 1) are optimized such that the nanopillars’ polarization conversion efficiencies are relatively high across the entire visible spectral range, which ensures broadband operation and efficient generation of POVs and PPBs. In addition, thanks to the high refractive index of the constituent material TiO_2_, the optical fields at different wavelengths are all confined within individual nanopillars (Supplementary Fig. 4). This makes the TiO_2_ nanopillar behave as a weakly coupled low-quality factor resonator, and thus validates the approximation of each nanopillar to be the local pixel of Jones matrix.

### Generalized PPBs generated by the metasurfaces

Based on the above principle, two TiO_2_ nanopillar metasurfaces (namely, MF1 and MF2) of area 90 μm × 90 μm are designed to generate two kinds of PPBs (PPB1 and PPB2). These two metasurfaces share the same device parameters: NA = 0.1, *f* = 200 μm, and *γ* = 1.2 at the design wavelength of *λ*_0_ = 530 nm. PPB1 generated by MF1 is the superposition of two POVs with topological charge numbers (*l*_*m*_,_1_ = 1, *l*_*n, 1*_ = 5) expressed by $\cos \,\frac{\alpha }{2}{e}^{i\beta /2}|{{\rm{POV}}}_{{\mathrm{R}}},l=1\rangle +\,\sin \,\frac{\alpha }{2}{e}^{-i\beta /2}|{{\rm{POV}}}_{{\mathrm{L}}},l=5\rangle$, while PPB2 generated by MF2 is the superposition of two POVs with topological charge numbers (${l}_{m,2}=5,{l}_{n,2}=10$) expressed by $\cos \,\frac{\alpha }{2}{e}^{i\beta /2}|{{\rm{POV}}}_{{\mathrm{R}}},l=5\rangle +\,\sin \,\frac{\alpha }{2}{e}^{-i\beta /2}|{{\rm{POV}}}_{{\mathrm{L}}},l=10\rangle$. The independent spatial phase profiles for LCP and RCP light encoded onto the metasurface are shown in Supplementary Fig. 5. According to these spatial phase profiles, the phase shifts (*δ*_*x*_, *δ*_*y*_) and rotation angle *θ* of the birefringent TiO_2_ nanopillars as a function of the spatial coordinates in the metasurface (MF1 and MF2) plane can be obtained and shown in Fig. 2e.

**Fig. 3:**
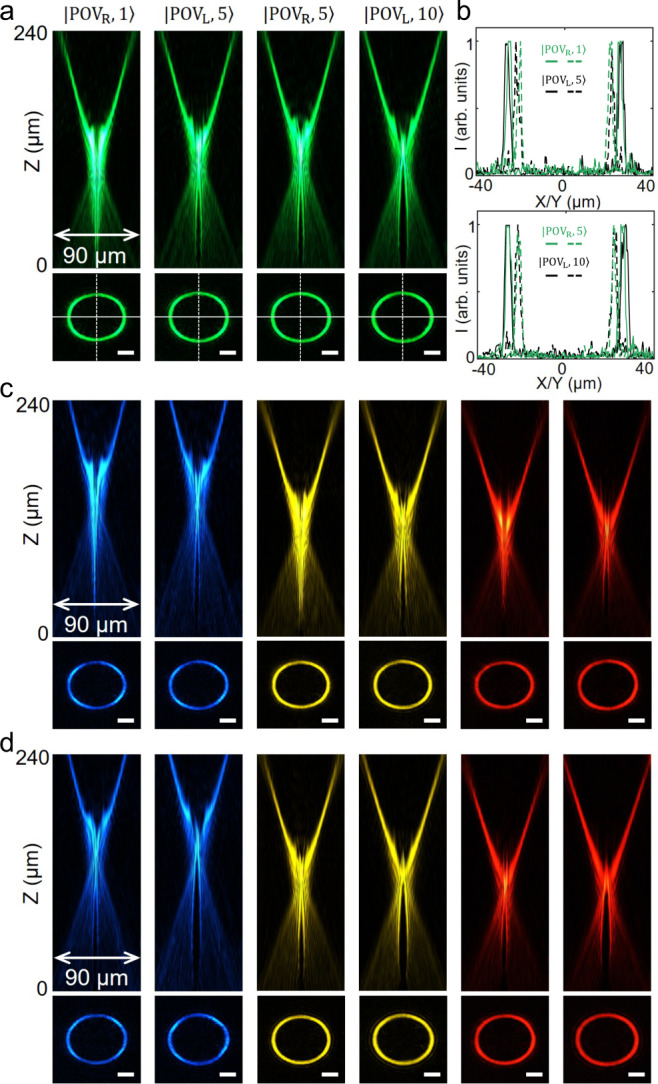
Characterization of metasurfaces for the generation of arbitrary POVs. **a** Top: measured intensity distributions of the four optical vortices with a linear scale in the *y–z* plane at a design wavelength of 530 nm: $|{{\rm{POV}}}_{{\mathrm{R}}},1\rangle$ and$|{{\rm{POV}}}_{{\rm{L}}},5\rangle$ corresponding to the metasurface MF1; $|{{\rm{POV}}}_{{\rm{R}}},5\rangle$ and $|{{\rm{POV}}}_{{\rm{L}}},10\rangle$ corresponding to the metasurface MF2. Bottom: measured annular intensity profiles of four optical vortices at the designed focal position *z* = 200 μm. Scale bar: 10 μm. **b** Normalized cross sections of the annular intensity profiles of the four optical vortices along the white dash and solid lines of **a** for MF1 (top) and MF2 (bottom). **c**, **d** Top: measured normalized intensity distributions of the optical vortices with a linear scale in the *y–z* plane at the wavelengths of 480 nm (blue), 580 nm (yellow) and 630 nm (red). Each wavelength corresponds to two orthogonally circular polarization optical vortices (**c**: $|{{\rm{POV}}}_{{\rm{R}}},1\rangle$ and $|{{\rm{POV}}}_{{\rm{L}}},5\rangle$ for metasurface MF1; **d**: $|{{\rm{POV}}}_{{\rm{R}}},5\rangle$ and $|{{\rm{POV}}}_{{\rm{L}}},10\rangle$ for metasurface MF2). Bottom: measured annular intensity profiles of optical vortices at the propagation distances *z* = 230 μm, 195 μm, and 180 μm corresponding to the wavelengths of 480 nm (blue), 580 nm (yellow) and 630 nm (red). Scale bar: 10 μm.

Figure 2f shows the optical microscopy image and scanning electron microscopy (SEM) images of MF1 (Top) and MF2 (Bottom). The metasurface fabrication details are given in the “Methods” section. Before the generation of PPBs, we first characterize the constituent spin-multiplexed optical vortices generated by the metasurfaces. By illuminating with RCP and LCP light generated from a supercontinuum laser attached to an acousto-optic tunable filter (AOTF) system, the in-plane (*x–y* plane) annular intensity distributions of the four generated optical vortices from MF1 $(|{{\rm{POV}}}_{{\mathrm{R}}},\,1\rangle ,|{{\rm{POV}}}_{{\mathrm{L}}},\,5\rangle )$ and MF2 $(|{{\rm{POV}}}_{{\mathrm{R}}},5\rangle ,|{{\rm{POV}}}_{{\mathrm{L}}},10\rangle )$ are captured at a step of 1 μm along the light propagation direction (*z*-axis). The top panel of Fig. 3a shows the spatial intensities in the *x–z* plane of the generated optical vortices at the wavelength of *λ*_0_ = 530 nm by stitching all the captured in-plane images together. It can be clearly seen that although the incident spin states and topological charges are all different for these two metasurfaces, the spatial patterns of the generated optical vortices along the propagation direction look very similar. The bottom panel of Fig. 3a shows the measured annular intensity profiles of four optical vortices at the designed focal position *z* = 200 μm. As expected, four in-plane elliptical intensity profiles are almost identical. To better quantitively evaluate the quality of the generated optical vortices, Fig. 3b shows the cross sections of in-plane intensity profiles extracted from Fig. 3a along the *x*- and the *y*-direction, respectively depicted by the white dashed and solid lines. At the designed wavelength of *λ*_0_ = 530 nm, the vertical radius *R*_⊥_, horizontal radius *R*_||_ and ellipticity factor *γ* = *R*_||_/*R*_⊥_ of the generated optical vortices are measured as following: for MF1, *R*_||_ = 24.3 μm, *R*_⊥_ = 19.9 μm, *γ* = 1.22 for RCP and *R*_||_= 25.1 μm, *R*_⊥_ = 20.9 μm, *γ* = 1.20 for LCP; For MF2, *R*_||_= 25.2 μm, *R*_⊥_ = 20.8 μm, *γ* = 1.21 for RCP and *R*_||_= 25.7 μm, *R*_⊥_ = 21.2 μm, *γ* = 1.21 for LCP. The measured results are very close to the theoretical values of *R*_||_ = 24 μm, *R*_⊥_ = 20 μm, *γ* = 1.20. These results explicitly imply that the intensity profiles of the optical vortices generated by the metasurfaces are insensitive to the topological charge numbers, proving that they are POVs and thus can be used for the generation of PPBs. The experimentally measured generation efficiencies, defined as the ratio of the optical intensity of generated POVs to the intensity of the incident light, range between 50 and 54% for both MF1 and MF2 (see Supplementary Table 2).

In addition to the designed wavelength of 530 nm, thanks to the relatively high polarization conversion efficiency of the optimized nanopillars across the entire visible region, the metasurfaces exhibit a broadband response (see Supplementary Note 5 for details of broadband operation principle) and can also operate efficiently at other wavelengths. Figure 3c, d show the experimentally captured intensity profiles of the optical vortices generated by MF1 and MF2 at wavelengths of 480 nm (blue), 580 nm (yellow) and 630 nm (red). From the intensity profiles in the *x–z* plane (top panel of Fig. 3c, d), as expected the focal length and NA of the metasurface are wavelength-dependent due to chromatic dispersion, however, the optical vortices still exhibit very consistent spatial intensity distributions at these different wavelengths. At positions *z* = 230 μm, 195 μm and 180 μm, respectively corresponding to blue, yellow and red light, the in-plane annular intensity patterns (bottom panel of Fig. 3c, d) are almost identical to the one measured in green (Fig. 3a). Therefore, the metasurfaces are still suitable for the generation of PBBs at other visible wavelengths. The cross sections of in-plane annular intensity profiles along *x*- and *y*-direction are given in Supplementary Fig. 11. The measured conversion efficiencies of these two metasurfaces for POVs at multiple wavelengths ranges from 31 to 45% (see Supplementary Table 2).

**Fig. 4:**
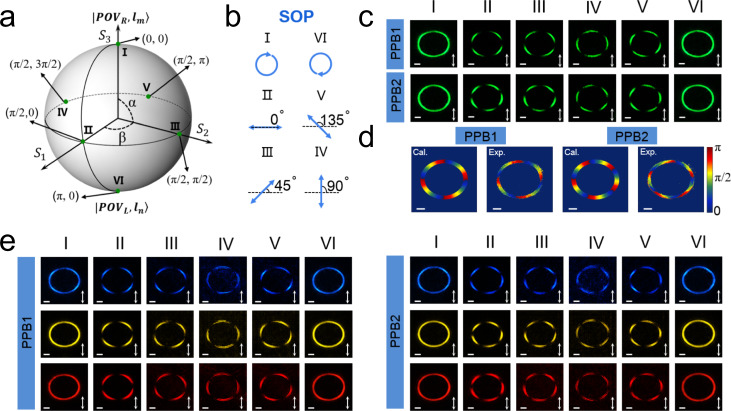
Evolutions of the metasurface-generated PPBs corresponding to the points on HyOPS. **a** The selected six points on HyOPS representing six states of PPBs generated successively by metasurface. **b** Six states of polarization (SOP) of the light incident on the metasurfaces are chosen to generate various states of PPBs corresponding to the coordinates in (**a**). **c** The measured annular intensity patterns of the output states corresponding to the points in **a** for PPB1 and PPB2 in the *x*–*y* plane after transmission through a vertical linear polarizer depicted by the white double arrow. These images are captured at the designed focal position *z* = 200 μm. Scale bar: 10 μm. **d** The calculated and measured polarization orientations and distributions of PPB1 and PPB2 corresponding to point II in (**a**). Note that the horizontal polarization orientation is defined as 0 rad. Scale bar: 10 μm. **e** Measured annular intensity patterns of the PPB1 and PPB2 in the *x*–*y* plane at the wavelengths of 480 nm (blue), 580 nm (yellow) and 630 nm (red) at a propagation distance of *z* = 230, 195, and 180  μm. These images are captured through a linear polarizer depicted by the white double arrow. Scale bar: 10 μm.

Next, to characterize these two metasurfaces for the generation of different PPBs, six points on the HyOPS are selected and their coordinates are given in Fig. 4a. The corresponding polarization states of light (Fig. 4b) incident on the metasurfaces are selected by rotating a quarter wave plate and a linear polarizer. The schematic of the experimental setup is illustrated in Supplementary Fig. 12. According to the topological charges of the constituent spin-multiplexed POVs, PPB1 and PPB2, generated from MF1 and MF2, respectively, have polarization order *p* and topological Pancharatnam charge *l*_*p*_ with ${p}_{1}=({l}_{m,1}-{l}_{n,1})/2=-2,{l}_{p,1}=({l}_{m,1}+{l}_{n,1})/2=3$ for PPB1, and ${p}_{2}=({l}_{m,2}-{l}_{n,2})/2=-2.5,{l}_{p,2}=({l}_{m,2}+{l}_{n,2})/2=7.5$ for PPB2. Figure 4c shows the experimentally measured intensity patterns of PPB1 and PPB2 in the *x–y* plane at the focal position *z* = 200 μm at a free-space wavelength of 530 nm. All the annular intensity patterns captured through a vertical linear polarizer for the two metasurfaces are of same contours, indicating that the radii of the generated PPBs are independent of sphere coordinate, polarization order and topological Pancharatnam charge. As the polarization order *p* determines the number of polarization rotations per round trip, the lobe number of the PPB pattern can be derived as 2|*p|* from the anisotropic polarization distribution through a vertical polarizer. As shown in the second column to fifth column (Fig. 4c), PPB1 and PPB2 are split into four and five lobes, respectively, which match well with the theoretical predictions of *p*_1_ = −2 and *p*_2_ = −2.5. In addition, we further confirm the anisotropic polarization distribution by Stokes polarimetry (see Supplementary Note 6 for details). The calculated and measured polarization distributions of the selected states corresponding to point II in Fig. 4a for the two kinds of PPBs are shown in Fig. 4d. It is clear that the polarization orientations rotate 4π and 5π per round trip for PPB1 and PPB2, respectively. The experimental results of these two PPBs agree well with the theoretical calculations (see Supplementary Note 7 for details and Supplementary Fig. 15 for calculated intensity patterns), except for a small rotation of the measured intensity and polarization patterns induced by the Gouy phase. In principle, the Gouy phase is dependent on the topological charge and can make the annular intensity pattern and polarization orientation distribution rotate by a small angle^48^. As a consequence, this simple and efficient method to generate PPBs also offers a potential solution to visualize the Gouy phase experimentally.

Besides the operation wavelength of 530 nm, Fig. 4e shows the experimental results for MF1 and MF2 illuminated at three other wavelengths across the visible region (480, 580, and 630 nm). The intensity patterns for these illumination wavelengths are captured at *z* = 230, 195, and 180 μm, respectively. As expected, these lobed patterns exhibit very similar morphological characteristics as the one at a wavelength of 530 nm. Therefore, these results explicitly show that the metasurfaces can achieve efficient and broadband generation of PPBs in the visible region.

### Optical information encryption

In the previous section, using a dielectric metasurface we have demonstrated the generation of PPBs that are independent of the topological charge number and polarization order. In principle, this approach encoding SAM and OAM states of light into PPBs in parallel channels can be employed for all-optical information encryption. For instance, various distinguishable PPBs with different polarization orders and ellipticities can be used to encode different double-digit hexadecimal numbers. For e.g., if the absolute value of polarization order |*p*| and ellipticity factor *γ* of the intensity pattern respectively denote the first and second digit of a double-digit hexadecimal number, and then a PPB represents a byte of data. As a result, the combination of 16 different values of |*p*| and *γ* for the PPBs can represent 256 hexadecimal numbers from 00 to FF. In our design, polarization order |*p*| ranging from 0 to 7.5 with an interval of 0.5 corresponds to the first hexadecimal digit from 0 to F, while ellipticity factor *γ* ranging from 0.5 to 2.0 with an interval of 0.1 represents the second hexadecimal digit from 0 to F. This code chart is given in Supplementary Table 3 and can be used to denote the characters of various encoding schemes.

**Fig. 5:**
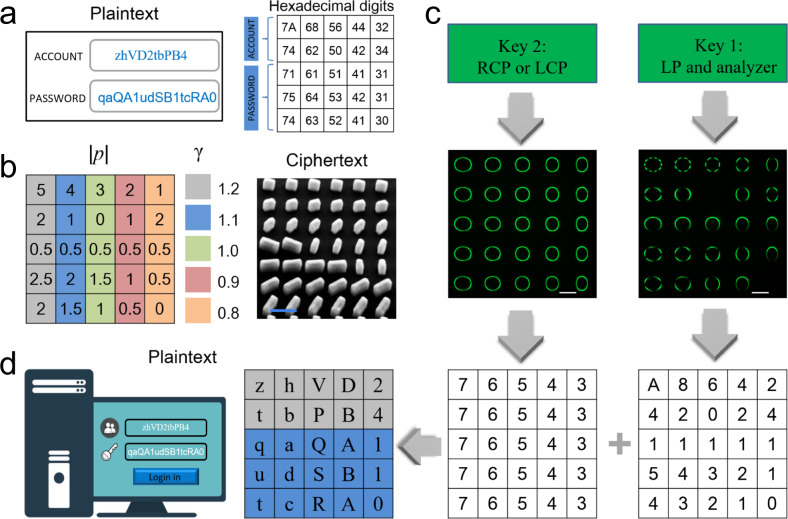
Proof-of-concept experimental demonstration of optical information encryption. **a** The plaintext message including a set of complex account number and password composed of different characters (left) are translated into the combinations of hexadecimal numbers (right) by User 1. **b** The design parameters of the 25 PPBs (left) and SEM image of a portion of the metasurface termed ciphertext (right). Scale bar: 500 nm. **c** User 2 captures two images with the two customized keys. According to the code chart, the first and second digits of these two-digit hexadecimal numbers are decrypted by User 2, respectively. Scale bar: 50 μm. **d** The hexadecimal number combination is decrypted as the plaintext message including the account number and password based on the ASCII.

During the encryption, two states of perfect Poincaré beams’ annular intensity images are combined to represent a double-digit hexadecimal number, which does not directly expose the encrypted information and increases the security. Anyone can access the original information only when he or she simultaneously possess all four indispensable hardware and software contents including metasurface device (ciphertext) as the encrypted information carrier, customized keys for the acquisition of beam intensities, code chart for decrypting numbers and character encoding system for mapping numbers to plaintext composed of various characters. Although someone may steal the metasurface device and keys and capture the two kind of intensity patterns, they cannot decode the correct hexadecimal numbers without a code chart. Crucially, the code chart and character encoding system can be self-defined and not fixed. Therefore, it is almost impossible to access the information without accurate one-to-one mapping relationship. As a proof-of-concept demonstration, here we use American Standard Code for Information Interchange (ASCII) to implement the optical encoding and decoding for information encryption. User 1 wants to send a set of high-security account number and password to User 2 and translates the plaintext into the combinations of ASCII hexadecimal numbers as illustrated in Fig. 5a. According to the hexadecimal numbers, 25 PPBs with the same *f* = 200 μm and NA = 0.1 but different values of |*p*| and *γ* are designed and encrypted on the metasurface, termed ciphertext (Fig. 5b). Then User 1 sends the metasurface sample and a customized key to User 2. With the illumination of horizontal linearly polarized (LP) light at a wavelength of 530 nm on the metasurface, an image of PPBs array is captured with the filtration of a vertical linear polarizer (right panel of Fig. 5c). Based on the code chart, the first hexadecimal digit can be determined by the lobe number of annular patterns. For instance, the first and last hexadecimal number in the text chart are respectively identified as “A” and “0”, on the basis of the fact that light beam is spilt into 10 lobes on the top left corner and completely blocked on the bottom right corner. On the other side, when the metasurface is illuminated with LCP or RCP light (left panel of Fig. 5c), another image of PPBs array without the filtration of a linear polarizer can be captured and the second hexadecimal digit can be determined by the ellipticity of annular patterns. For example, the first and last hexadecimal number in the text chart are respectively identified as “7” and “3” based on the annular beam with *γ* = 1.2 on the top left corner and another annular beam with *γ* = 0.8 on the bottom right corner. On the basis of this decoding rule, 25 double-digit hexadecimal numbers can be easily decrypted by the image identification. According to the ASCII (Supplementary Table 4), a customized program then converts the decrypted numbers into a string of ASCII characters including the account number and password information for User 2 to login on a computer (Fig. 5d). In addition, this metasurface device also operates over a broad spectral range in the visible (see Supplementary Fig. 16) as evident from the intensity of PPB array at wavelengths of 480, 580, and 630 nm which show similar patterns as the ones for the designed wavelength of 530 nm.

## Discussion

In conclusion, we have proposed and experimentally demonstrated broadband generation of perfect Poincaré beams via a single-layer dielectric metasurface for visible light. By changing the spin angular momentum of incident light, the metasurface device with high spatial sampling resolution can generate spin-multiplexed perfect optical vortices with arbitrary orbital angular momentum. Via spatial superposition of two perfect optical vortices, different perfect Poincaré beams, whose total angular momenta are described by the hybrid-order Poincaré sphere, can be generated. Furthermore, based on the angular momenta states encoded within the generated perfect Poincaré beams, a proof-of-concept experimental demonstration for optical information encryption is implemented. We envision that this work will inspire creation of ultracompact flat nanophotonic elements for efficient generation and control of structured beams and further promote their practical applications, such as optical communication, optical encryption, optical data storage, and quantum information sciences.

## Methods

### Numerical simulations

Full-wave numerical simulations are performed using the finite-difference time-domain (FDTD) technique. Rectangular TiO_2_ nanopillars with a fixed height of 600 nm are sitting on a fused-silica substrate with a lattice constant of 450 nm. The incident plane-wave is polarized along *x*- or *y*- axes and illuminates the nanopillars from the substrate side. Along *x* and *y* axes, periodic boundary conditions are applied and perfectly matched layer (PML) boundary condition is used in the *z* direction. The phase shifts (*P*_*x*_ and *P*_*y*_) and power transmission (*T*_*x*_ and *T*_*y*_) (see Supplementary Fig. 1) are obtained by parameter sweeping of the in-plane dimensions (*D*_*x*_ and *D*_*y*_) of the nanopillars by varying them between 50 nm and 350 nm at an interval of 5 nm.

### Sample fabrication

Fused-silica substrates were coated with a monolayer of hexamethyldisilazane and a layer of 600 nm thick positive-tone electron beam (e-beam) resist. In order to eliminate the charging effect during e-beam lithography, a layer of 10 nm thick aluminum (Al) was deposited on the samples by thermal evaporation. Then e-beam lithography was performed at an accelerating voltage of 100 kV and beam current of 2 nA. Afterward, the samples were developed in hexyl-acetate for 120 s and then coated with TiO_2_ using atomic layer deposition (ALD) at a low temperature of 90 °C. The overcoated TiO_2_ layer was etched by the inductively-coupled-plasma reactive ion etching. Finally, the samples were exposed to UV irradiation and followed by soaking in n-methyl-2-pyrrolidone.

## Supplementary information

Supplementary Information

## Data Availability

The data that support the findings of this study are available from the corresponding author upon reasonable request.
