# Supplementary material for: Broadband generation of perfect Poincaré beams via dielectric spin-multiplexed metasurface
Source: Nat Commun. 2021 Apr 13;12:2230. doi: 10.1038/s41467-021-22462-z (PMC8044217; doi:10.1038/s41467-021-22462-z)
Supplement: Supplementary file 1 — Supplementary Information [file 41467_2021_22462_MOESM1_ESM.pdf]

# Supplementary for Broadband generation of perfect Poincaré beams via dielectric spin-multiplexed metasurface

Mingze Liu[1,2], Pengcheng Huo[1,2], Wenqi Zhu[3,4], Cheng Zhang[5], Si Zhang[1,2], Maowen Song[1,2], Song Zhang[1,2], Qianwei Zhou[1,2], Lu Chen[3,4], Henri J. Lezec[3], Amit Agrawal[3,4], Yanqing Lu[1,2] and Ting Xu[1,2]

1. National Laboratory of Solid-State Microstructures, Jiangsu Key Laboratory of Artificial Functional Materials, College of Engineering and Applied Sciences, Nanjing University, Nanjing 210093, China
2. Collaborative Innovation Center of Advanced Microstructures, Nanjing 210093, China
3. Physical Measurement Laboratory, National Institute of Standards and Technology, Gaithersburg, Maryland 20899, United States
4. Maryland NanoCenter, University of Maryland, College Park, Maryland 20899, United States
5. School of Optical and Electronic Information, Wuhan National Laboratory for Optoelectronics, Huazhong University of Science and Technology, Wuhan 430074, China.

**Supplementary Note 1. Derivation of POV using the Fourier transform of higher-order Bessel beam.**

In theory, a POV can be generated by the Fourier transform of a higher-order Bessel beam[1]. Because it is nontrivial to generate an ideal Bessel beam experimentally, we consider a Bessel-Gaussian beam which can be generated by passing a Gaussian beam through an axicon. The complex field amplitude of a Bessel-Gaussian beam with uniform circular polarization in the cylindrical coordinate system $(\rho, \phi, z)$ is expressed as[2]:

$$\boldsymbol{E}(\rho, \phi, z) = J_l(k_r\rho)\exp(il\phi)\exp(ik_z z)\exp\left(-\frac{\rho^2}{\omega_g}\right)\begin{bmatrix}1\\ \pm i\end{bmatrix} \quad (1)$$

where $J_l$ is an $l$-th order Bessel function of the first kind, $k_r$ and $k_z$ are the radial and longitudinal wavevectors and $\omega_g$ is the waist of the Gaussian beam. The Fourier transform of the Bessel-Gaussian beam can be implemented by an optical lens. The Fourier transformation of the optical field $\boldsymbol{E}(\rho, \phi)$ into $\boldsymbol{E}(r, \varphi)$ is expressed as[3]:

$$\boldsymbol{E}(r, \varphi) = \frac{k}{i2\pi f}\int_0^{\infty}\int_0^{2\pi}\boldsymbol{E}(\rho, \phi)\rho\exp\left[-\frac{ik\rho r\cos(\varphi-\phi)}{f}\right]d\rho\, d\phi \quad (2)$$

where $f$ is the focal length of lens. By substituting eq. 1 into eq. 2, the complex field amplitude of the POV in the focal plane is obtained as:

$$\boldsymbol{E}(r, \varphi) = \frac{\omega_g i^{l-1}}{\omega_\gamma}\exp(il\varphi)I_l\left(\frac{2R_\gamma r}{{\omega_\gamma}^2}\right)\exp\left(\frac{-r^2-{R_\gamma}^2}{\omega_\gamma^2}\right)\begin{bmatrix}1\\ \pm i\end{bmatrix} \quad (3)$$

where $I_l$ is an $l$-th order modified Bessel function of the first kind, $r = \sqrt{(x)^2+(y\gamma)^2}$, $\varphi = \mathrm{actan}(\gamma y/x)$, $R_\gamma = \gamma k_r f/k$, $\gamma$ is a scale factor and controls the ellipticity of POV, ($x$, $y$) are Cartesian coordinates in the rear focal plane of the lens, $R_\gamma = \gamma k_r f/k$, $k = 2\pi/\lambda$ is the wave vector, $f$ is the focal length of the lens, $k_r$ can be controlled by the numerical aperture of the axicon NA, and $\omega_\gamma = 2\gamma f/k\omega_g$ is waist of Gaussian beam at the rear focal plane of the lens. For small $\omega_\gamma$ at the focus of the lens and large $R_\gamma$, $I_l\left(\frac{2R_\gamma r}{{\omega_\gamma}^2}\right)$ can be approximated as $\exp\left(\frac{2R_\gamma r}{\omega_\gamma^2}\right)$. Eq. 3 can be rewritten as:

$$\boldsymbol{E}(r, \varphi) = \frac{\omega_g i^{l-1}}{\omega_\gamma}\exp(il\varphi)\exp\left(\frac{-(r-R_\gamma)^2}{\omega_\gamma^2}\right)\begin{bmatrix}1\\ \pm i\end{bmatrix} \quad (4)$$

Note that the amplitude of the POV is shaped by a Gaussian function with a maximum intensity at $r = R_{\gamma}$. The radius of the POV along the vertical and horizontal directions are $R_{\perp} = f\mathrm{NA}$ and $R_{\parallel} = \gamma f\mathrm{NA}$, which are both independent of topological charges.

**Supplementary Note 2. Phase distributions of the metasurface for generation of PPBs**

As shown in the main text, the PPBs are composed of orthogonal circularly polarized POVs, and the POVs can be generated by the Fourier transformation of a higher-order Bessel-Gaussian beam. In the experiments, a Gaussian beam can become POV after successively passing through a spiral phase plate, axicon and Fourier transformation lens. A metasurface can implement this multielement multifunctional process for either RCP or LCP incident light by satisfying the superposition of the phase distributions of a spiral phase plate $\varphi_{spiral}(x,y)$, an axicon $\varphi_{axicon}(x,y)$ and a Fourier transformation lens $\varphi_{lens}(x,y)$ which are expressed as:

$$\varphi_{spiral}(x,y) = l \bullet \arctan(y/\gamma x) \tag{5}$$

$$\varphi_{axicon}(x,y) = -\frac{2\pi}{\lambda}\sqrt{(\gamma x)^2 + (y)^2} \bullet \mathrm{NA} \tag{6}$$

$$\varphi_{lens}(x,y) = -\frac{\pi}{\lambda f}(x^2 + y^2) \tag{7}$$

where $(x, y)$ is the geometric coordinate of the metasurface and $l = l_m$ or $l_n$ for RCP or LCP light respectively. Thus the phase profile $\varphi_{meta}(x,y) = \varphi_1(x,y)$ or $\varphi_2(x,y)$ encoded respectively on the metasurface for RCP or LCP light is described as:

$$\varphi_{meta}(x,y) = \varphi_{spiral}(x,y) + \varphi_{axicon}(x,y) + \varphi_{lens}(x,y) \tag{8}$$

**Supplementary Note 3. Derivation of the Jones matrix $J$ and its eigenvalues and**

**eigenvectors:**

An arbitrary polarized light beam normally incident onto the metasurface can be decomposed into RCP and LCP spin eigenstates with corresponding two-dimensional (2D) Jones vectors given by: $|\mathrm{LCP}\rangle = \begin{bmatrix} 1 \\ i \end{bmatrix}$ and $|\mathrm{RCP}\rangle = \begin{bmatrix} 1 \\ -i \end{bmatrix}$. In order to generate two completely different POVs represented by two poles of the HyOPS, the metasurface is required to provide two independent spatial phase profiles $\varphi_1(x,y)$ and $\varphi_2(x,y)$ corresponding to $|\mathrm{LCP}\rangle$ and $|\mathrm{RCP}\rangle$, where $\varphi_1(x,y)$ and $\varphi_2(x,y)$ are the superposition of the phase distributions of the spiral phase plate, axicon and Fourier transform lens. That is, for LCP incident light, the metasurface can implement the transformation: $|\mathrm{LCP}\rangle \rightarrow |\mathrm{POV}_R, l_m\rangle$. The output beam $|\mathrm{POV}_R, l_m\rangle$ will have opposite handedness compared to the incident beam. Similarly, the same metasurface can also transform RCP incident light to a different POV with LCP state and topological charge of $l_n$: $|\mathrm{RCP}\rangle \rightarrow |\mathrm{POV}_L, l_n\rangle$. The metasurface can be described by a Jones matrix $J(x,y)$ which simultaneously satisfies $J(x,y)|\mathrm{LCP}\rangle = \exp[i\varphi_1(x,y)]|\mathrm{RCP}\rangle$ and $J(x,y)|\mathrm{RCP}\rangle = \exp[i\varphi_2(x,y)]|\mathrm{LCP}\rangle$ at each point $(x, y)$. Matrix inversion of these two equations results in:

$$J(x,y) = \begin{bmatrix} \exp[i\varphi_1(x,y)] & \exp[i\varphi_2(x,y)] \\ -i\exp[i\varphi_1(x,y)] & i\exp[i\varphi_2(x,y)] \end{bmatrix} \begin{bmatrix} 1 & 1 \\ i & -i \end{bmatrix}^{-1} \tag{9}$$

The required Jones matrix is calculated as:

$$J(x,y) = \begin{bmatrix} \frac{\exp[i\varphi_1(x,y)]+\exp[i\varphi_2(x,y)]}{2} & \frac{i\exp[i\varphi_2(x,y)]-i\exp[i\varphi_1(x,y)]}{2} \\ \frac{i\exp[i\varphi_2(x,y)]-i\exp[i\varphi_1(x,y)]}{2} & \frac{-\exp[i\varphi_1(x,y)]-\exp[i\varphi_2(x,y)]}{2} \end{bmatrix} \tag{10}$$

Due to symmetric and unitary conditions, $J(x,y)$ can be written in a standard form $J(x,y) = R\Lambda R^{-1}$, where $R$ is a real unitary matrix and $\Lambda$ is a diagonal matrix. By solving the characteristic equation of $J(x,y)$, its eigenvalues are calculated as:

$$\lambda_1 = e^{i\frac{1}{2}[\varphi_1(x,y)+\varphi_2(x,y)]} \qquad \lambda_2 = e^{i\frac{1}{2}[\varphi_1(x,y)+\varphi_2(x,y)]-i\pi} \tag{11}$$

and eigenvectors as:

$$V_1 = [\cos\theta \quad \sin\theta]^T \qquad V_2 = [-\sin\theta \quad \cos\theta]^T \tag{12}$$

where $\theta = \frac{1}{4}[\varphi_1(x,y) - \varphi_2(x,y)]$. Thus, the Jones matrix can be rewritten in terms of its

eigenvectors and eigenvalue as:

$$J(x,y) = R\Lambda R^{-1} = [V_1 \quad V_2]\begin{bmatrix}\lambda_1 & 0 \\ 0 & \lambda_2\end{bmatrix}[V_1 \quad V_2]^{-1} \tag{13}$$

Since the matrix $J(x,y)$ operates in the linear polarization basis, the diagonal matrix $\Lambda$ determines the phase shifts $\delta_x = \frac{1}{2}[\varphi_1\,(x,y) + \varphi_2\,(x,y)]$ and $\delta_y = \frac{1}{2}[\varphi_1\,(x,y) + \varphi_2\,(x,y)] - \pi$ along the two symmetry axes of the linearly birefringent element. Meanwhile, the matrix $R$ corresponds to a rotation matrix for $\Lambda$ and determines the rotation angle $\theta = \frac{1}{4}[\varphi_1\,(x,y) - \varphi_2\,(x,y)]$ of the fast axis of the birefringent element in the *x*-*y* plane. In order to implement $J(x,y)$, a series of sub-wavelength nanopillars are designed to provide the required phase shifts ($\delta_x$, $\delta_y$) covering an entire 2π phase range and satisfying the orientation angle ($\theta$) requirement at any point ($x$, $y$) of the metasurface.

**Supplementary Note 4. Calculation of polarization conversion efficiency of the unit cells.**

For an arbitrary polarized light $|E_{in}\rangle$ incident on the metasurface unit cell, the transmitted light $|E_{out}\rangle$ can be expressed as[4]:

$$|E_{out}\rangle = \eta_E|E_{in}\rangle + \eta_R e^{i2\theta}|\text{RCP}\rangle + \eta_L e^{-i2\theta}|\text{LCP}\rangle \quad (14)$$

where $\eta_E = \frac{1}{2}(T_x + T_y e^{i\xi})$ , $\eta_R = \frac{1}{2}(T_x - T_y e^{i\xi})\langle E_{in}|\text{LCP}\rangle$ and $\eta_L = \frac{1}{2}(T_x - T_y e^{i\xi})\langle E_{in}|\text{RCP}\rangle$ represent the transmission coefficients of different polarization orders and $\theta$ is the orientation of the fast axis of the metasurface unit cell. Here, $\langle E_{in}|\text{RCP or LCP}\rangle$ is an inner product. $T_x$ and $T_y$ are the spectral transmission coefficients of the unit-cell for light polarized parallel and perpendicular to the fast axis and $\xi$ is the phase difference between $T_x$ and $T_y$. For an incident light with circular polarization ($|\text{LCP}\rangle$ or $|\text{RCP}\rangle$), the polarization conversion efficiency ${\eta_R}^2$ or ${\eta_L}^2$ can be obtained according to the eq. 14 as:

$${\eta_R}^2 = \left|\frac{1}{2}(T_x - T_y e^{i\xi})\langle E_{in}|\text{LCP}\rangle\right|^2 \quad (15)$$

$${\eta_L}^2 = \left|\frac{1}{2}(T_x - T_y e^{i\xi})\langle E_{in}|\text{RCP}\rangle\right|^2 \quad (16)$$

**Supplementary Note 5. Broadband response for the proposed metasurface**

As for broadband response, the proposed metasurface composed of sub-wavelength nanopillars has a broadband phase modulation of two orthogonal circular polarization states. The geometric phase is wavelength-insensitive and each metasurface nanopillar with same orientation angle can impart same geometric phase value at various wavelengths. Meanwhile, the broadband-response propagation phases with same linear gradients imparted by a group of eight selected metasurface nanopillars can always cover the entire 0 to $2\pi$ phase range at various wavelengths. The two broadband phase modulations ensure that the proposed metasurface can completely offer RCP or LCP phase profiles with very similar contours at wavelengths of 480 nm, 530 nm, 580 nm and 630 nm, respectively. Therefore, the metasurface-offered broadband RCP and LCP phase profiles can achieve broadband generation of orthogonal circular polarized perfect vortices and perfect Poincaré beams. However, due to the existence of chromatic dispersion, the beam size and divergence angles of perfect vortices and perfect Poincaré beams are not identical for various incident wavelengths.

To further analyze and explain broadband generation of perfect Poincaré beams achieved here, we must quantificationally clarify the phase response achieved from the metasurface for two orthogonal circular polarization states at different incident wavelengths. In terms of the metasurface nanopillars' phase modulation used in this paper, the geometric phase depends only on the geometry of the pathway through the anisotropy transforming the light wave and is wavelength-insensitive. Specifically, each metasurface nanopillar acting as a half waveplate with same orientation angle ($\theta$) can offer same PB phase ($\varphi_{PB} = 2\theta$) of the output wavefront at various wavelengths. Then, we simulated the geometric phases of eight nanopillars chosen for this study with a certain orientation angle at different wavelengths. As an example, Supplementary Fig. 6 shows the simulated geometric phases of eight selected nanopillars with same orientation angle of $\pi/2$ when illuminating circularly polarized light at wavelengths of 480 nm, 530 nm, 580 nm and 630 nm. The simulated geometric phases of cross-polarized output wavefronts at various wavelengths are consistent with the theoretical values (Supplementary Fig. 6) of $\pi$ and thus the geometric phase is wavelength-insensitive.

As for the propagation phase, it is accumulated through light wave propagating in the nanopillar behaving as a truncated waveguide, which can be expressed as:

$$\varphi_{PG}(\lambda) = (2\pi/\lambda)\, n_e H \tag{17}$$

where $\lambda$ is the wavelength of incident light, $n_e$ is the effective refractive index of propagating optical mode which depends on the lateral size of the $TiO_2$ nanopillar and its surroundings medium (air), and $H$ is the height of the nanopillar. Because the refractive index of $TiO_2$ nanopillar is relatively flat in the visible between wavelengths of 450 nm and 650 nm (Supplementary Fig. 7), the effective refractive index $n_e$ basically depends on the size of $TiO_2$ nanopillar and is approximately wavelength-independent. Theoretically, the propagation phase shift $\varphi_{constant}(\lambda)$ of a nanopillar between the designed wavelength ($\lambda_0$) and another wavelength ($\lambda$) is proportional to $(\frac{1}{\lambda}-\frac{1}{\lambda_0})$. Supplementary Fig. 8a shows the theoretical propagation phase offered by the set of eight nanopillars chosen for this study at different incident wavelengths of 480 nm, 580 nm and 630 nm. These phases, all offer the same linear gradient at various wavelengths can cover the requisite full 2π phase range. To further verify the above theoretical analysis, we simulated and calculated the phase of complex polarization conversion coefficient of $(t_x - t_y)/2$ for the eight selected nanopillars at different incident wavelength of 480 nm, 530 nm, 580 nm and 630 nm (Supplementary Fig. 8b), where $t_x$ and $t_y$ represent complex transmission coefficients for *x*- and *y*- polarized light incident on the nanopillar, respectively. The phase part of complex polarization conversion coefficient which is the propagation phase offered by the eight nanopillars can cover the entire 0 to 2π phase range at the design wavelength of 530nm as well as at other wavelengths of 480 nm, 580 nm and 630 nm. The simulated propagation phases accord closely with the theoretical propagation phases. In addition, the relatively constant propagation phase differences of each nanopillar between the designed wavelength ($\lambda_0$= 530 nm) and other incident wavelengths (λ =480 nm, 580 nm or 630 nm) can't disorganize the entire in-plane phase distributions for RCP and LCP light offered by the proposed metasurface at various wavelengths (discussed in next paragraph).

Meanwhile, the phase profiles imparted by the broadband metasurface for RCP or LCP light at a specific wavelength can be expressed as:

$$\varphi_{RCP/LCP}(\lambda, x, y) = \varphi_{PG}(\lambda, x, y) \pm \varphi_{PB}(x, y) \quad (18)$$

where $\varphi_{PG}(\lambda, x, y)$ is the propagation phase accrued upon propagation through a $TiO_2$ nanopillar for different wavelengths at any point $(x, y)$ of the metasurface, $\varphi_{PB}(x, y)$ is geometric phase and equals $2\theta(x, y)$ where $\theta(x, y)$ is the in-plane rotation angle of a $TiO_2$ nanopillar at any point $(x, y)$ of the metasurface. Considering phase modulation mechanism, the exact geometric phase and propagation phase imparted by each metasurface nanopillar at different wavelengths, as an example, the theoretical and simulated phase profiles for both RCP and LCP light offered by a metasurface for generation of two orthogonal circularly polarized perfect vortices at wavelengths of 480 nm, 530 nm, 580 nm and 630 nm are given in Supplementary Fig. 9. The in-plane phase profiles for RCP and LCP light are normalized to 0 to 2π range. The metasurface parameters are as follows: two topological charges $l_m$ of 5 and $l_n$ of 10, ellipticity factor $\gamma$ of 1.2, focal length $f$ of 200 μm, numerical aperture NA of 0.1 and designed wavelength $\lambda_0$ of 530 nm. The theoretical phase profiles (Supplementary Fig. 9a) for RCP and LCP light at each wavelength maintains the same contour, respectively, which arises from the same $\varphi_{constant}(\lambda)$ for the eight nanopillars at a specific wavelength $\lambda$. The simulated phase profiles (Supplementary Fig. 9b) for RCP and LCP light are well matched with the theoretical results. This indicates that the proposed metasurface can achieve broadband generation of two orthogonal circularly polarized perfect vortices because of its broadband phase response for RCP and LCP light.

In order to see the phase shifts between design wavelength and other wavelengths more clearly, the cross sections of these in-plane phase profiles are extracted from Supplementary Fig. 9 along $x$-axis depicted by dashed white lines and unwrapped, as shown in Supplementary Fig. 10. The simulated results also agree well with the theoretical results.

**Supplementary Note 6. Stokes polarimetry of PPBs and spherical wave interferometry**

The polarization state of PPBs on the HyOPS is mapped by representing the Stokes parameters in the spherical Cartesian coordinates. Stokes polarimetry is implemented by measuring a series of intensity distributions to obtain the values of Stokes parameters expressed as[5]:

$$S_0 = I_0 + I_{90} \tag{19}$$

$$S_1 = I_0 - I_{90} \tag{20}$$

$$S_2 = I_{45} - I_{135} \tag{21}$$

$$S_3 = I_R - I_L \tag{22}$$

where $I_0$, $I_{45}$, $I_{90}$ and $I_{135}$ are the intensities of PPBs after transmission through a rotated linear polarizer oriented at 0°, 45°, 90° and 145° with respect to the $x$-axis respectively. $I_R$ and $I_L$ are the intensities of PPBs after transmission through a right and a left circular polarizer, respectively. In order to reduce the numbers of measurements, $I_{135}$ and $I_L$ can be expressed as: $I_{135} = I_0 + I_{90} - I_{45}$; $I_L = I_0 + I_{90} - I_R$. Therefore, all the Stokes parameters can be obtained by measuring four intensity distributions ($I_0$, $I_{45}$, $I_{90}$ and $I_R$) through the PPBs. The spherical coordinates ($\alpha$, $\beta$) of the points on the HyOPS can be determined by the Stokes parameters: $\alpha = \arccos\left(\frac{S_3}{S_0}\right)$ and $\beta = \arctan\left(\frac{S_2}{S_1}\right)$. Thus, the spatial distribution of polarization orientation angle $\Theta$ for the PPB described by any point on the HyOPS can be obtained as:

$$\Theta = \frac{1}{2}\arctan\left(\frac{S_2}{S_1}\right) \tag{23}$$

In order to analyze the polarization distribution of the PPBs in experiment, a linear polarizer and a quarter waveplate are inserted at the front of the camera to measure these four intensities distributions ($I_0$, $I_{45}$, $I_{90}$ and $I_R$). In addition, in order to accurately distinguish state A and state F in Figure 1a, Stokes polarimetry and spherical wave interferometry should be performed on the two beams, respectively. First, a measurement of four intensities ($I_0$, $I_{45}$, $I_{90}$ and $I_R$) of the two beams after transmission through a quarter waveplate and a linear polarizer must be performed. According to these intensities (Supplementary Fig. 13), one can calculate the spherical coordinates ($\alpha$, $\beta$) of the two beams corresponding to the point on the HyOPS: for

state A, the spherical coordinates is (0, 0) which corresponds to RCP perfect vortex; for state F, the spherical coordinates is (π, 0) which corresponds to LCP perfect vortex. Furthermore, to determine the topological charge of the perfect vortex, the RCP or LCP spherical wave is interfered with RCP or LCP perfect vortex. The simulated interference patterns are shown in Supplementary Fig. 14. According to the number of lobes, one can determine the topological charges of RCP and LCP perfect vortex to be 5 and 10, respectively. Therefore, using the measurement sequence outlined above, one can distinguish state A from state F and accurately determine the polarization distribution and topological charges of the two beams. As for the measurement of other states on the HyOPS, Stokes polarimetry only needs to be implemented for these beams.

**Supplementary Note 7. Intensity calculation for PPBs with superpositions of POVs**

According to the Eq. 4, the magnitude of a POV with topological charge *l* is expressed as:

$$E_l(r,\varphi) = \frac{\omega_g i^{l-1}}{\omega_\gamma} \exp(il\varphi) \exp\left(\frac{-(r-R_\gamma)^2}{\omega_\gamma^2}\right) \tag{24}$$

As shown in the main text, the PPB is the superposition of two orthogonal circular polarization POVs. For an arbitrary polarization state of light incident on the metasurface device, the output light is a superposition of the form given by eq. 1. The intensity of the output PPB corresponding to point ($\alpha, \beta$) on the HyOPS is calculated by the following equation:

$$\langle U_N | U_N \rangle = \left| \cos(\frac{\alpha}{2}) e^{i\beta/2} E_{l_m}(r,\varphi) \begin{bmatrix} 1 \\ -i \end{bmatrix} + \sin(\frac{\alpha}{2}) e^{-i\beta/2} E_{l_n}(r,\varphi) \begin{bmatrix} 1 \\ i \end{bmatrix} \right|^2 \tag{25}$$

where $E_{l_m}(r,\varphi)$ and $E_{l_n}(r,\varphi)$ denote the magnitudes of the RCP and LCP POVs with topological charges $l_m$ and $l_n$ given by eq. 24. Cos $(\frac{\alpha}{2})$ and sin $(\frac{\alpha}{2})$ determine the weights of RCP and LCP POV and $\beta$ is the relative phase shift between them. The metasurface-generated PPB can be characterized by a linear polarizer with orientation angle $\chi$ from the $x$ axis. The intensity of the PPB after transmission through a linear polarizer is expressed as:

$$T_{LP} = |[\begin{matrix} \cos^2\chi & \cos\chi\sin\chi \\ \cos\chi\sin\chi & \sin^2\chi \end{matrix}] \bullet |U_N\rangle|^2 \tag{26}$$

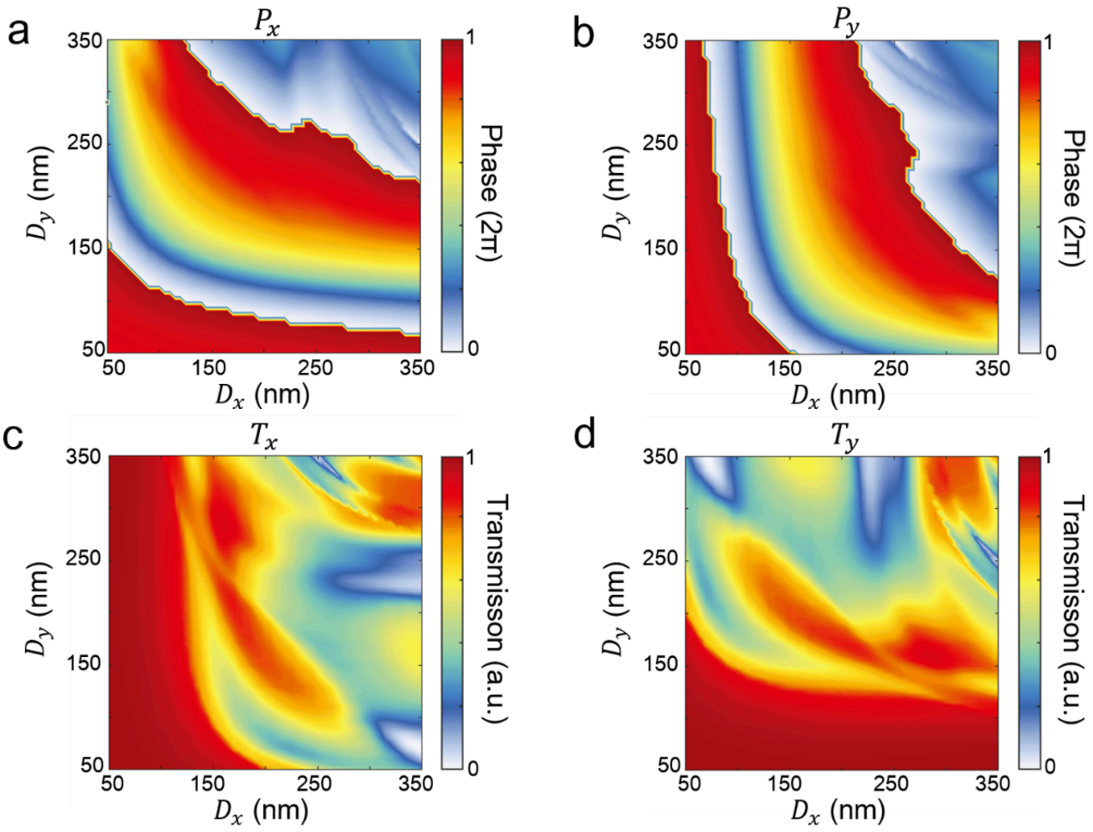


**Supplementary Fig. 1.** Simulated phase shifts (**a**. $P_x$; **b**. $P_y$) and power transmission coefficients (**c**. $T_x$; **d**. $T_y$) for *x*- and *y*- polarized light as functions of the rectangular nanopillar diameters ($D_x$ and $D_y$) at a free-space wavelength of 530 nm.

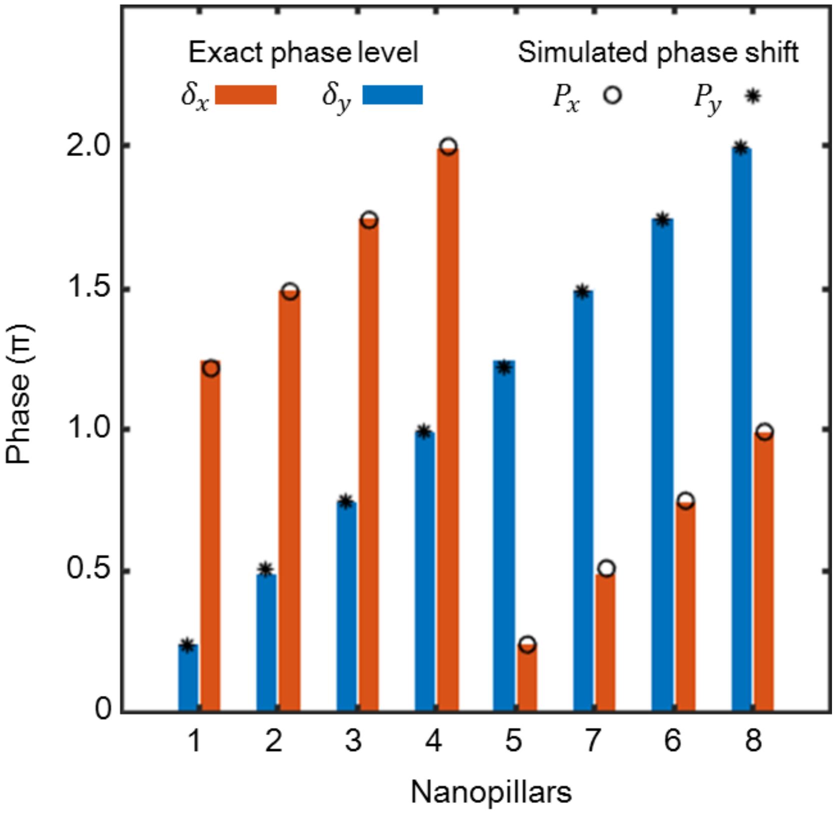


**Supplementary Fig. 2**. Eight level of discrete phase in the full range [0, 2π] for $\delta_x$ and $\delta_y$ (depicted by red and blue bars, respectively) and simulated phase shifts $P_x$ and $P_y$ (depicted by circle and star symbols, respectively) of eight selected TiO2 nanopillars at the designed wavelength of 530 nm. Each phase level corresponds to a specific TiO2 nanopillar which is also a half-wave plate.

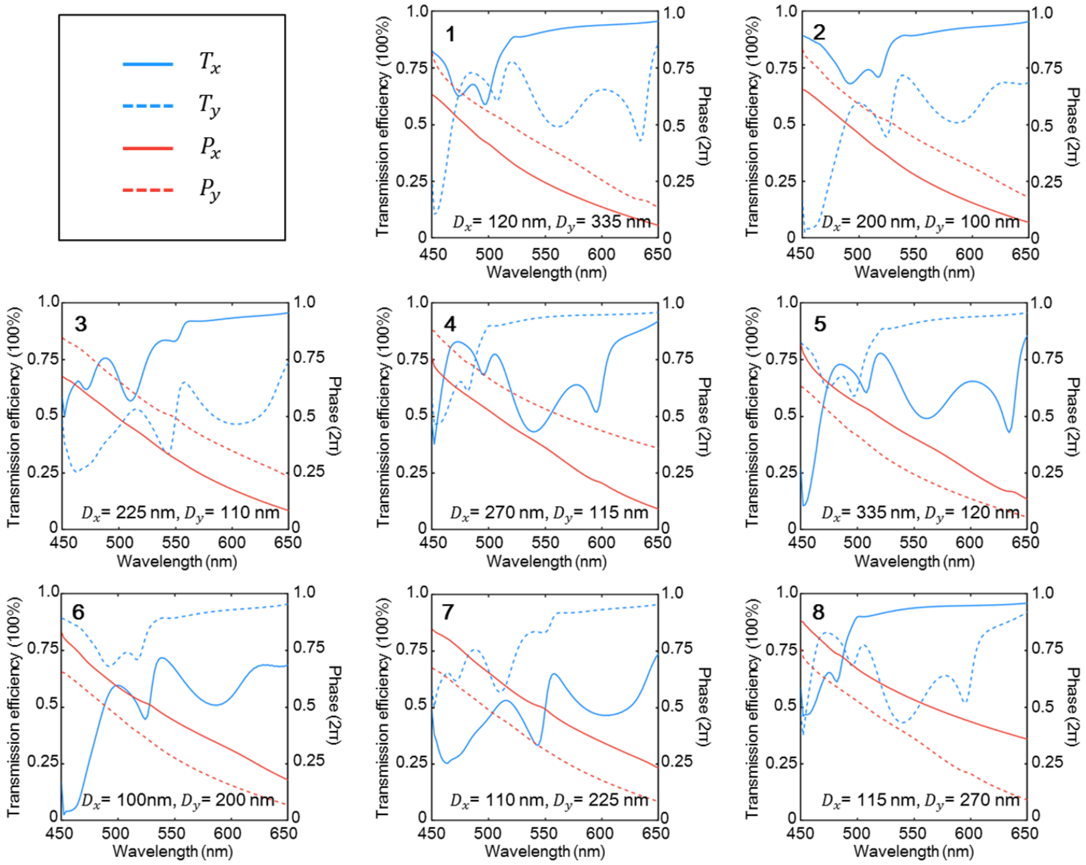


**Supplementary Fig. 3.** Wavelength dependence of power transmission efficiency ($T_x$ and $T_y$) and phase shifts ($P_x$ and $P_y$) of eight selected nanopillars (numbered 1-8) for *x*- and *y*-polarized light in the visible.

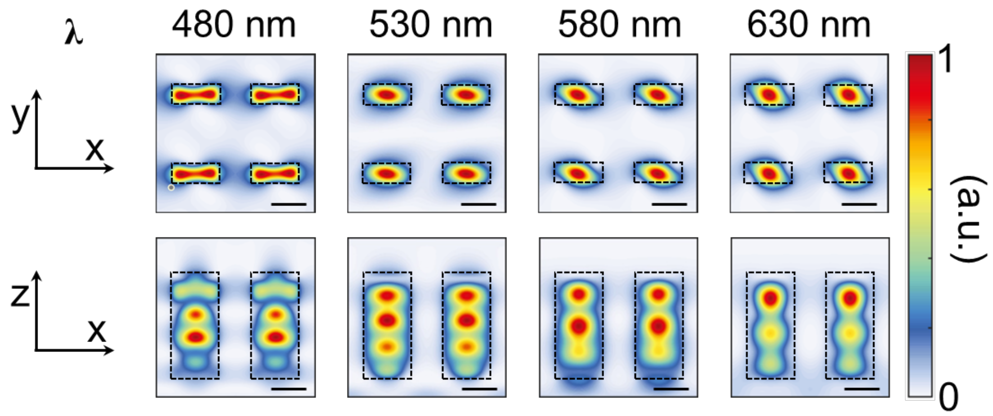


**Supplementary Fig. 4.** Simulated normalized magnetic energy density of the nanopillar array for various incident wavelengths. The dimensions of each nanopillar are $D_x$ = 270 nm and $D_y$ = 115 nm. Dashed black lines depict the boundaries of the nanopillars (top: *x*–*y* cross-section; bottom: *x*–*z* cross-section). Scale bar: 200 nm.

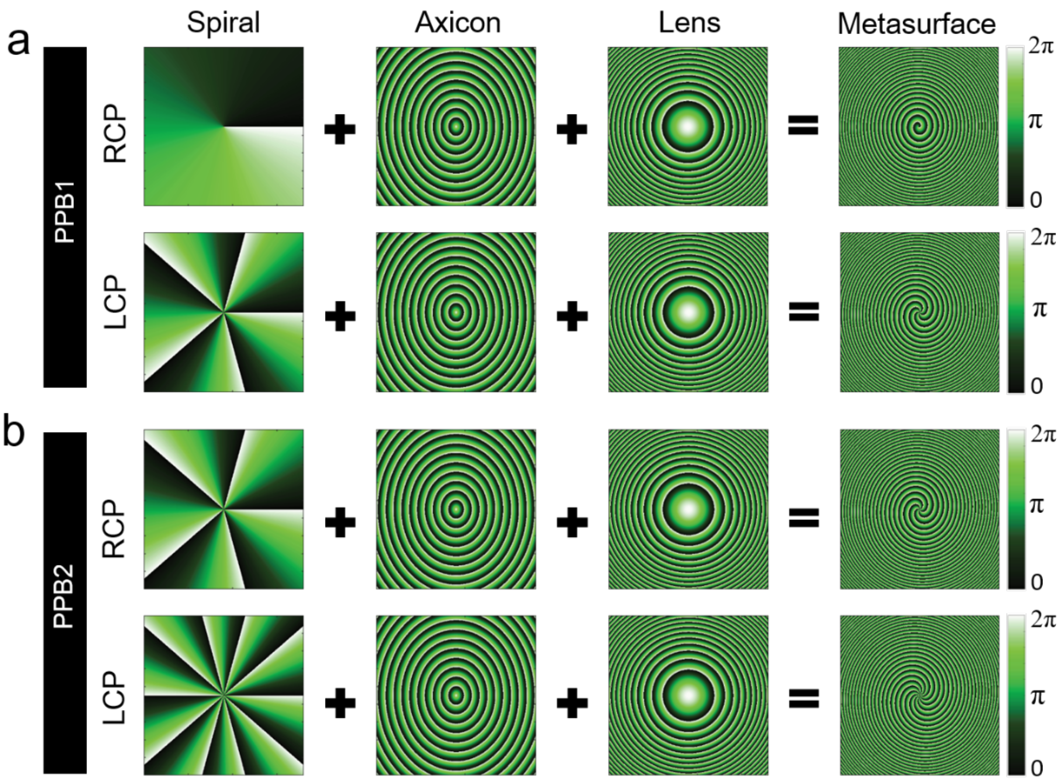


**Supplementary Fig. 5.** Phase distributions encoded on the metasurface for the genration of PPBs. **a** The metasurface MF1 for the generation of PPB1 should import the phase profile $\varphi_1(x,y)$ with $l_m=1$ for RCP light and another phase profile $\varphi_2(x,y)$ with $l_m=5$ for LCP light. **b** The metasurface MF2 for the generation of PPB2 should import the phase profile $\varphi_1(x,y)$ with $l_m=5$ for RCP light and another phase profile $\varphi_2(x,y)$ with $l_m=10$ for LCP light. The phase profiles $\varphi_1(x,y)$ and $\varphi_2(x,y)$ for MF1 and MF2 are the superposition of phase distributions of the spiral phase plate, axicon and lens and share the same values of NA = 0.1, $\gamma$ =1.2 and $f$ = 200 μm.

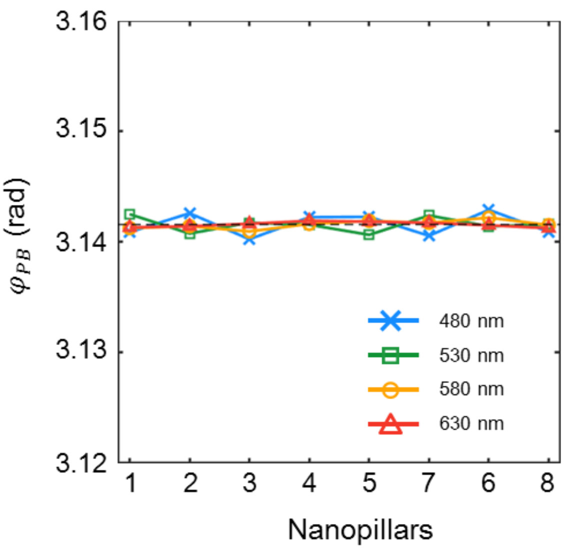


**Supplementary Fig. 6.** Theoretical and simulated geometric phases of eight selected nanopillars with same orientation angle of π/2. The black dashed line denotes the same theoretical geometric phase values of π at wavelengths of 480 nm, 530 nm, 580 nm and 630 nm. The cross, square, circle and triangle denote the simulated geometric phases at different incident wavelengths of 480 nm (blue), 530 nm (green), 580 nm (yellow) and 630 nm (red), respectively.

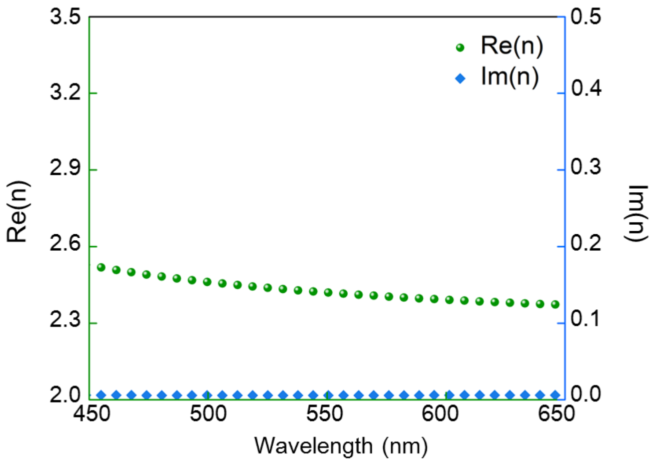


**Supplementary Fig. 7.** Complex refractive index of atomic layer deposition (ALD) amorphous titanium dioxide ($TiO_2$). The real (green circles) and imaginary (blue squares) part of the complex refractive index as a function of wavelength are measured by spectroscopic ellipsometry.

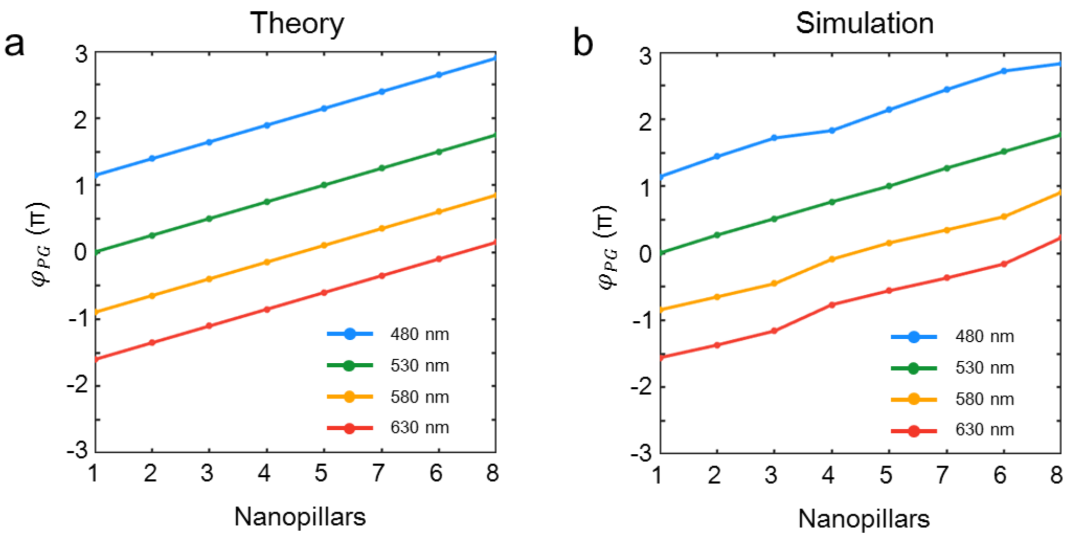


**Supplementary Fig. 8.** Theoretical (a) and simulated (b) propagation phases generated by 8-step nanopillars at different incident wavelength of 480 nm (blue), 530 nm (green), 580 nm (yellow) and 630 nm (red) can cover the entire 0 to 2π phase range.

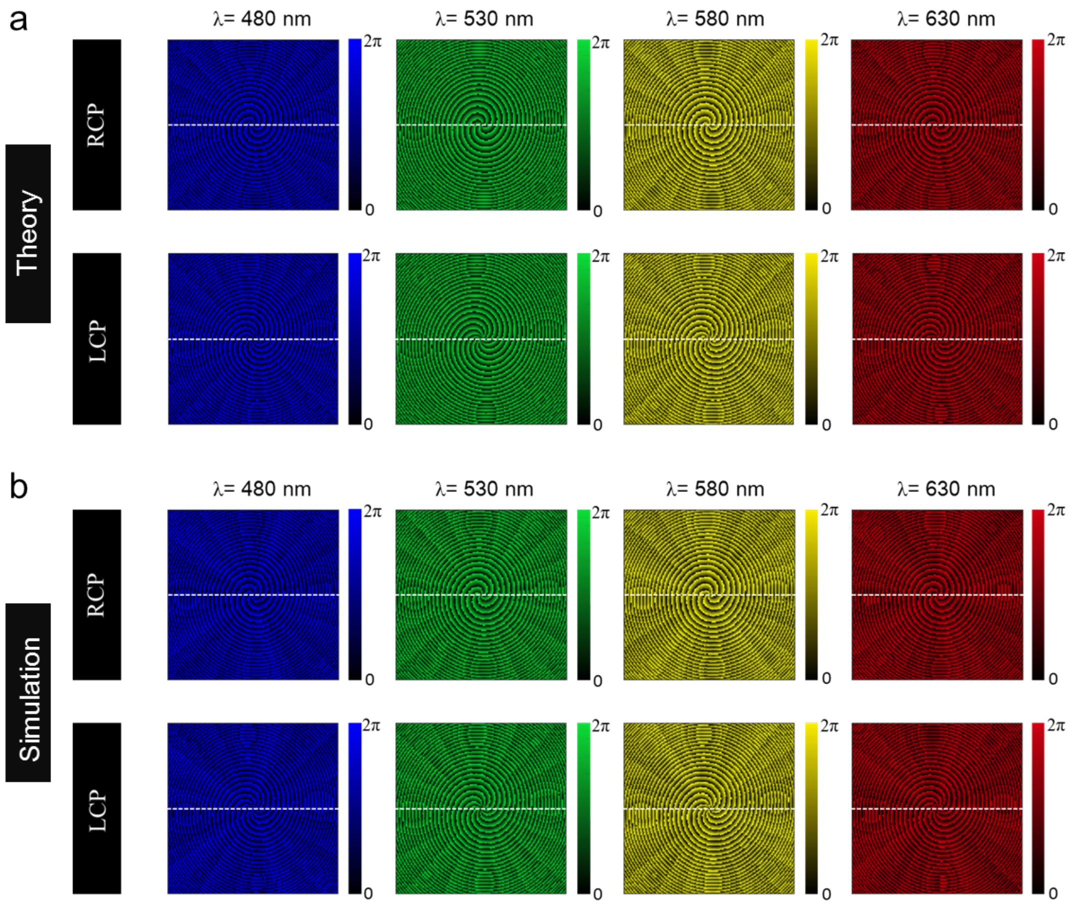


**Supplementary Fig. 9.** Theoretical and simulated in-plane phase profiles for RCP and LCP light offered by a broadband metasurface for generation of two orthogonal circularly polarized perfect vortices at different incident wavelength of 480 nm (blue), 530 nm (green), 580 nm (yellow) and 630 nm (red).

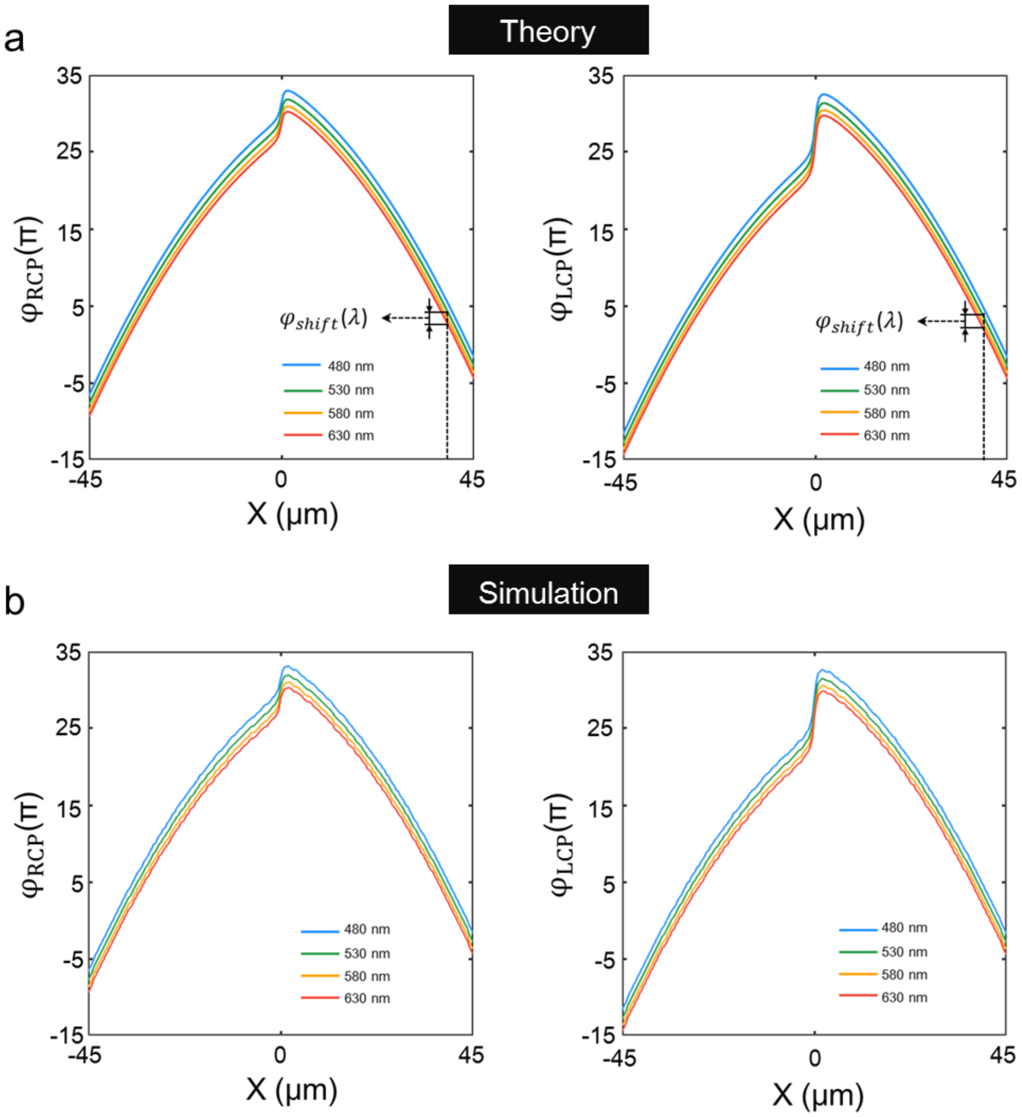


**Supplementary Fig. 10.** Cross sections of theoretical (a) and simulated (b) metasurface-imparted in-plane phase profiles along $x$-axis for RCP and LCP light at the incident wavelengths of 480 nm, 530 nm, 580 nm and 630 nm. $\varphi_{shift}(\lambda)$ is a wavelength-dependent and coordinate-independent constant phase shift and in accord with the $\varphi_{constant}(\lambda)$.

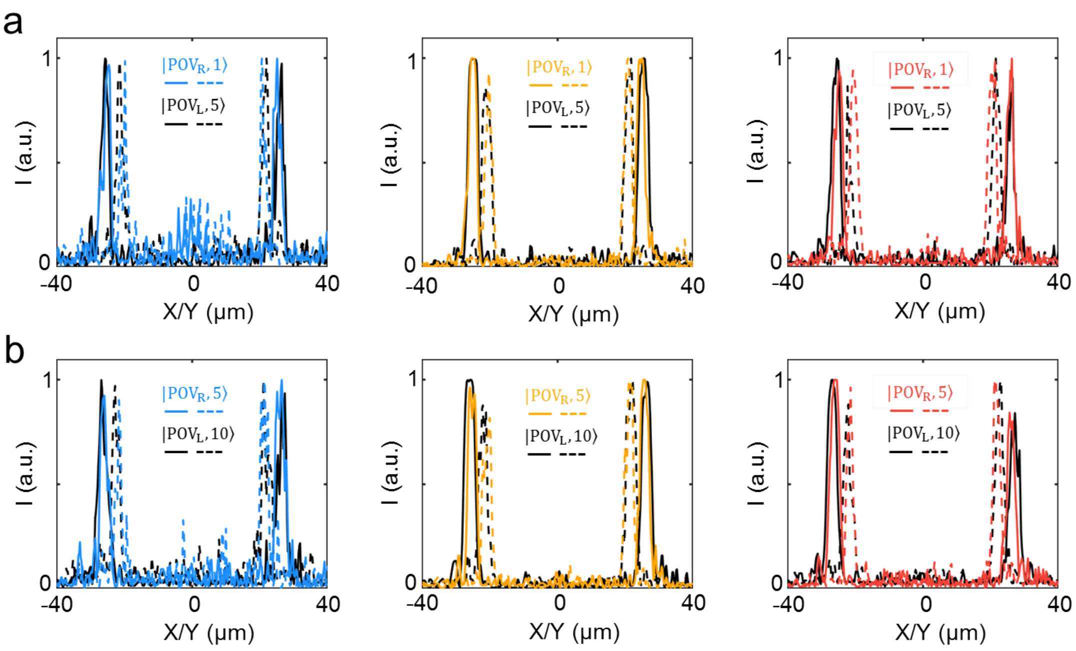


**Supplementary Fig. 11.** Normalized cross-sections of the annular intensity profiles of the optical vortices along $x$- and $y$- directions at wavelengths: 480 nm (blue), 580 nm (yellow) and 630 nm (red) at propagation distance of $z$ = 230 μm, 195 μm and 180 μm, respectively for MF1 (**a**) and MF2 (**b**).

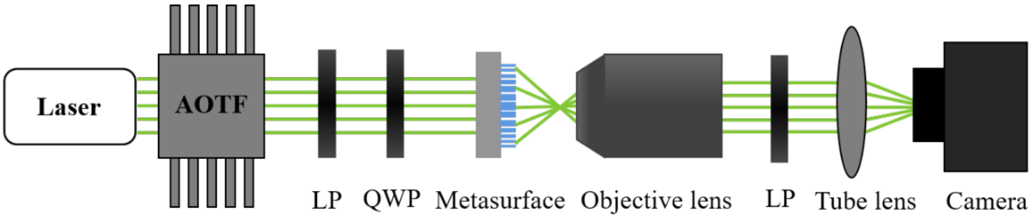


**Supplementary Fig. 12.** Experimental setup for measuring metasurface devices generating arbitrary states of PPBs on the HyOPS. A supercontinuum laser filtered by an acousto-optic tunable filter (AOTF) system passing through a linear polarizer (LP) and quarter waveplate (QWP) are converted into the desired polarization lights and incident on the metasurfaces. The transmitted lights are captured after filtering through a linear polarizer (LP).

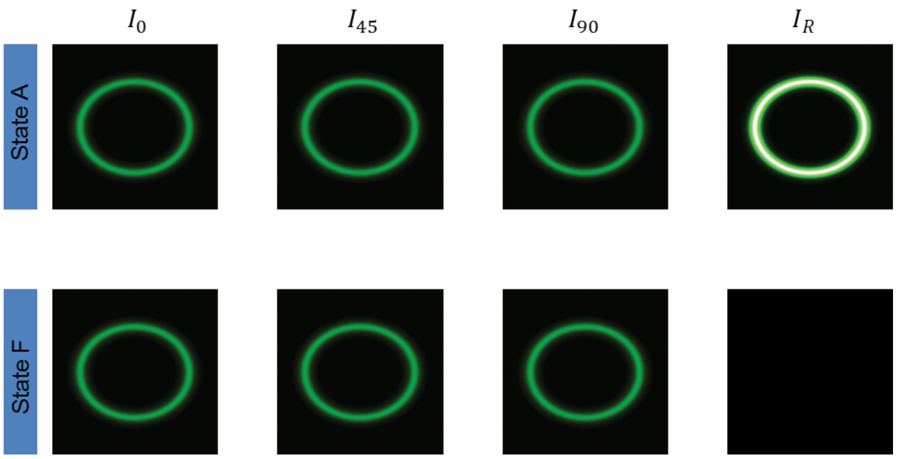


**Supplementary Fig. 13.** Calculated intensities of $I_0$, $I_{45}$, $I_{90}$ and $I_R$ for state A and state F of the PPB in Fig. 1a performed by Stokes polarimetry.

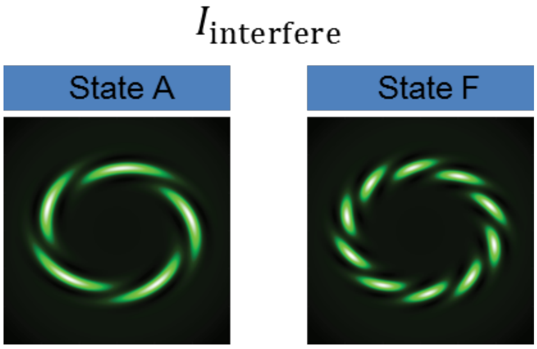


**Supplementary Fig. 14.** Simulated interference patterns of state A and state F of the PPB in Fig. 1a. The two beam of state A and state F are with topological charges of $l_m = 5$ and $l_n = 10$, respectively.

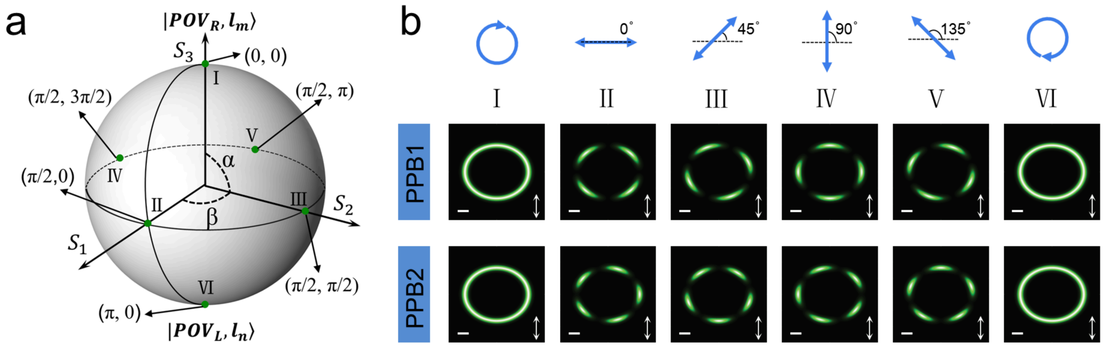


**Supplementary Fig. 15.** Calculated intensities of the PPBs corresponding to the points on the HyOPS. **a** The six selected points on HyOPS represent the six states of PPB. **b** Top: the polarization states of the incident light. Bottom: the calculated annular intensity patterns corresponding to the points in **a** for PPB1 and PPB2 after transmission through a vertical linear polarizer depicted by the white double arrow. Scale bar: 10 μm.

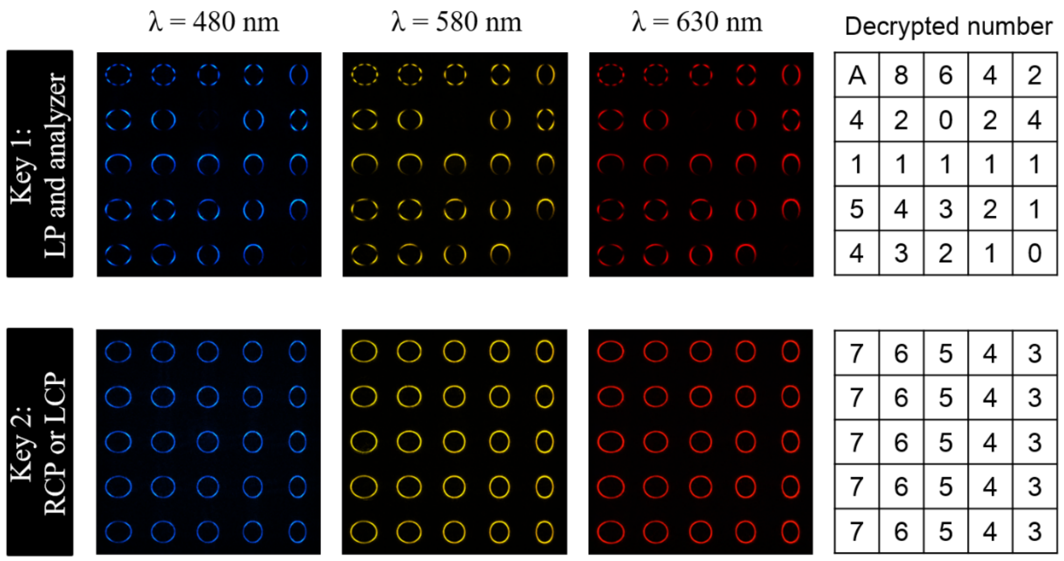


**Supplementary Fig. 16.** Measured images with two customized keys at the wavelengths of 480 nm (blue), 580 nm (yellow) and 630 nm (red) at a propagation distance of $z$ = 230 μm, 195 μm and 180 μm, respectively. According to the code chart, the first and second digits of two-digit hexadecimal numbers can also be decrypted.

**Supplementary Table 1.** The geometrical parameters of the eight nanopillars selected to cover the entire $2\pi$ phase range.

| Number | Dx (nm) | Dy (nm) |
|---|---|---|
| 1 | 120 | 335 |
| 2 | 200 | 100 |
| 3 | 225 | 110 |
| 4 | 270 | 115 |
| 5 | 335 | 120 |
| 6 | 100 | 200 |
| 7 | 110 | 225 |
| 8 | 115 | 270 |

**Supplementary Table 2.** The conversion efficiencies of the fabricated metasurfaces for generation of POVs.

| Metasurface | 480 nm | 530 nm | 580 nm | 630 nm |
|---|---|---|---|---|
| MF 1 $\lvert \mathrm{POV_R}, l_1 \rangle$ | 35% ± 2% | 50% ± 2% | 42% ± 3% | 39% ± 2% |
| MF 1 $\lvert \mathrm{POV_L}, l_5 \rangle$ | 33% ± 2% | 54% ± 4% | 45% ± 3% | 40% ± 3% |
| MF 2 $\lvert \mathrm{POV_R}, l_5 \rangle$ | 31% ± 1% | 53%± 2% | 42%± 3% | 39%± 2% |
| MF 2 $\lvert \mathrm{POV_L}, l_{10} \rangle$ | 34% ± 3% | 51%±4% | 39%± 2% | 42%± 3% |

**Supplementary Table 3.** Code chart of 256 hexadecimal numbers from 00 to FF represented by 256 different kinds of PPBs. Here, $s_0$ to $s_{15}$ denote the sizes of PPBs depending on the $\gamma$ ranging from 0.5 to 2.0 and $p_0$ to $p_{15}$ denote the lobe numbers of PPBs depending on the $|p|$ ranging from 0 to 7.5.

| | $p_0$ | $p_1$ | $p_2$ | $p_3$ | $p_4$ | $p_5$ | $p_6$ | $p_7$ | $p_8$ | $p_9$ | $p_{10}$ | $p_{11}$ | $p_{12}$ | $p_{13}$ | $p_{14}$ | $p_{15}$ |
|---|---|---|---|---|---|---|---|---|---|---|---|---|---|---|---|---|
| $s_0$ | 00 | 01 | 02 | 03 | 04 | 05 | 06 | 07 | 08 | 09 | 0A | 0B | 0C | 0D | 0E | 0F |
| $s_1$ | 10 | 11 | 12 | 13 | 14 | 15 | 16 | 17 | 18 | 19 | 1A | 1B | 1C | 1D | 1E | 1F |
| $s_2$ | 20 | 21 | 22 | 23 | 24 | 25 | 26 | 27 | 28 | 29 | 2A | 2B | 2C | 2D | 2E | 2F |
| $s_3$ | 30 | 31 | 32 | 33 | 34 | 35 | 36 | 37 | 38 | 39 | 3A | 3B | 3C | 3D | 3E | 3F |
| $s_4$ | 40 | 41 | 42 | 43 | 44 | 45 | 46 | 47 | 48 | 49 | 4A | 4B | 4C | 4D | 4E | 4F |
| $s_5$ | 50 | 51 | 52 | 53 | 54 | 55 | 56 | 57 | 58 | 59 | 5A | 5B | 5C | 5D | 5E | 5F |
| $s_6$ | 60 | 61 | 62 | 63 | 64 | 65 | 66 | 67 | 68 | 69 | 6A | 6B | 6C | 6D | 6E | 6F |
| $s_7$ | 70 | 71 | 72 | 73 | 74 | 75 | 76 | 77 | 78 | 79 | 7A | 7B | 7C | 7D | 7E | 7F |
| $s_8$ | 80 | 81 | 82 | 83 | 84 | 85 | 86 | 87 | 88 | 89 | 8A | 8B | 8C | 8D | 8E | 8F |
| $s_9$ | 90 | 91 | 92 | 93 | 94 | 95 | 96 | 97 | 98 | 99 | 9A | 9B | 9C | 9D | 9E | 9F |
| $s_{10}$ | A0 | A1 | A2 | A3 | A4 | A5 | A6 | A7 | A8 | A9 | AA | AB | AC | AD | AE | AF |
| $s_{11}$ | B0 | B1 | B2 | B3 | B4 | B5 | B6 | B7 | B8 | B9 | BA | BB | BC | BD | BE | BF |
| $s_{12}$ | C0 | C1 | C2 | C3 | C4 | C5 | C6 | C7 | C8 | C9 | CA | CB | CC | CD | CE | CF |
| $s_{13}$ | D0 | D1 | D2 | D3 | D4 | D5 | D6 | D7 | D8 | D9 | DA | DB | DC | DD | DE | DF |
| $s_{14}$ | E0 | E1 | E2 | E3 | E4 | E5 | E6 | E7 | E8 | E9 | EA | EB | EC | ED | EE | EF |
| $s_{15}$ | F0 | F1 | F2 | F3 | F4 | F5 | F6 | F7 | F8 | F9 | FA | FB | FC | FD | FE | FF |

**Supplementary Table 4.** A portion of American Standard Code for Information Interchange. HEX denote the hexadecimal numbers and ABBR/CHAR denote the abbreviation of control character or displayable character.

| HEX | ABBR/ CHAR | HEX | ABBR/ CHAR | HEX | ABBR/ CHAR | HEX | ABBR/ CHAR | HEX | ABBR/ CHAR | HEX | ABBR/ CHAR |
|---|---|---|---|---|---|---|---|---|---|---|---|
| 20 | (Space) | 30 | 0 | 40 | @ | 50 | P | 60 | ` | 70 | p |
| 21 | ! | 31 | 1 | 41 | A | 51 | Q | 61 | a | 71 | q |
| 22 | " | 32 | 2 | 42 | B | 52 | R | 62 | b | 72 | r |
| 23 | # | 33 | 3 | 43 | C | 53 | S | 63 | c | 73 | s |
| 24 | $ | 34 | 4 | 44 | D | 54 | T | 64 | d | 74 | t |
| 25 | % | 35 | 5 | 45 | E | 55 | U | 65 | e | 75 | u |
| 26 | & | 36 | 6 | 46 | F | 56 | V | 66 | f | 76 | v |
| 27 | ' | 37 | 7 | 47 | G | 57 | W | 67 | g | 77 | w |
| 28 | ( | 38 | 8 | 48 | H | 58 | X | 68 | h | 78 | x |
| 29 | ) | 39 | 9 | 49 | I | 59 | Y | 69 | i | 79 | y |
| 2A | * | 3A | : | 4A | J | 5A | Z | 6A | j | 7A | z |
| 2B | + | 3B | ; | 4B | K | 5B | [ | 6B | k | 7B | { |
| 2C | , | 3C | < | 4C | L | 5C | \ | 6C | l | 7C | \| |
| 2D | - | 3D | = | 4D | M | 5D | ] | 6D | m | 7D | } |
| 2E | . | 3E | > | 4E | N | 5E | ^ | 6E | n | 7E | ~ |
| 2F | / | 3F | ? | 4F | O | 5F | _ | 6F | o | 7F | DEL |

## Supplementary References

[1] Ostrovsky, A., Rickenstorff-Parrao, C., Arrizón, V. Generation of the "perfect" optical vortex using a liquid-crystal spatial light modulator. Opt. Lett. 38, 534 (2013).

[2] Gori, F., Guattari, G., Padovani, C. Bessel-Gauss beam. Opt. Commun. 64, 491(1987).

[3] Goodman, J. Introduction to Fourier Optics (Roberts & Company, 2004).

[4] Mongia, R. K., Ittipiboon, A. Theoretical and experimental investigations on rectangular dielectric resonator antennas. IEEE Trans. Antenn. Propag. 45, 1348–1356 (1997).

[5] Schaefer, B., Collett, E., Smyth, R., Barrett, D., Fraher, B. Measuring the Stokes polarization parameters. Am. J. of Phys. 75(2), 163-168 (2007).
